# Local Innate Markers and Vaginal Microbiota Composition Are Influenced by Hormonal Cycle Phases

**DOI:** 10.3389/fimmu.2022.841723

**Published:** 2022-03-25

**Authors:** Cindy Adapen, Louis Réot, Natalia Nunez, Claude Cannou, Romain Marlin, Julien Lemaître, Léo d’Agata, Emmanuel Gilson, Eric Ginoux, Roger Le Grand, Marie-Thérèse Nugeyre, Elisabeth Menu

**Affiliations:** ^1^ Université Paris-Saclay, Inserm, Commissariat à l'énergie Atomique et aux énergies Alternatives (CEA), Center for Immunology of Viral, Auto-Immune, Hematological and Bacterial Diseases [IMVA-HB/Infectious Disease Models and Innovative Therapies (IDMIT)], Fontenay-aux-Roses, France; ^2^ Life&Soft, Fontenay-aux-Roses, France; ^3^ Mucosal Immunity and Sexually Transmitted Infection Control (MISTIC) Group, Department of Virology, Institut Pasteur, Paris, France

**Keywords:** inflammation, menstrual cycle, female reproductive tract (FRT), cytokines, blood compartment, neutrophils, vaginal microbiota

## Abstract

**Background:**

The female reproductive tract (FRT) mucosa is the first line of defense against sexually transmitted infection (STI). FRT environmental factors, including immune-cell composition and the vaginal microbiota, interact with each other to modulate susceptibility to STIs. Moreover, the menstrual cycle induces important modifications within the FRT mucosa. Cynomolgus macaques are used as a model for the pathogenesis and prophylaxis of STIs. In addition, their menstrual cycle and FRT morphology are similar to women. The cynomolgus macaque vaginal microbiota is highly diverse and similar to dysbiotic vaginal microbiota observed in women. However, the impact of the menstrual cycle on immune markers and the vaginal microbiota in female cynomolgus macaques is unknown. We conducted a longitudinal study covering three menstrual cycles in cynomolgus macaques. The evolution of the composition of the vaginal microbiota and inflammation (cytokine/chemokine profile and neutrophil phenotype) in the FRT and blood was determined throughout the menstrual cycle.

**Results:**

Cervicovaginal cytokine/chemokine concentrations were affected by the menstrual cycle, with a peak of production during menstruation. We observed three main cervicovaginal neutrophil subpopulations: CD11b^high^ CD101^+^ CD10^+^ CD32a^+^, CD11b^high^ CD101^+^ CD10^-^ CD32a^+^, and CD11b^low^ CD101^low^ CD10^-^ CD32a^-^, of which the proportion varied during the menstrual cycle. During menstruation, there was an increase in the CD11b^high^ CD101^+^ CD10^+^ CD32a^+^ subset of neutrophils, which expressed higher levels of CD62L. Various bacterial taxa in the vaginal microbiota showed differential abundance depending on the phase of the menstrual cycle. Compilation of the factors that vary according to hormonal phase showed the clustering of samples collected during menstruation, characterized by a high concentration of cytokines and an elevated abundance of the CD11b^high^ CD101^+^ CD10^+^ CD32a^+^ CD62L^+^ neutrophil subpopulation.

**Conclusions:**

We show a significant impact of menstruation on the local environment (cytokine production, neutrophil phenotype, and vaginal microbiota composition) in female cynomolgus macaques. Menstruation triggers increased production of cytokines, shift of the vaginal microbiota composition and the recruitment of mature/activated neutrophils from the blood to the FRT. These results support the need to monitor the menstrual cycle and a longitudinal sampling schedule for further studies in female animals and/or women focusing on the mucosal FRT environment.

## 1 Introduction

Heterosexual transmission from males to females is among the major routes of sexually transmitted infections (STIs) and occurs mainly through the female reproductive tract (FRT). The FRT is composed of a wide array of environmental factors, such as a mucus layer, secreted IgA/IgG, vaginal microbiota, local immune cells, an epithelium barrier, and soluble factors, such as antimicrobial peptides, cytokines, and chemokines (1). These factors all interact with each other to modulate susceptibility to pathogen invasion. They are affected by hormonal variations, which are involved in modifying the composition of the vaginal microbiota and the regulation of innate and adaptive immune responses within the FRT and peripheral blood (1–4). The menstrual cycle in women is composed of four phases: menstruation and the follicular phase (i.e., proliferative phase), ovulation phase, and luteal phase (i.e., secretory phase). Two main groups of hormones regulate the menstrual cycle: pituitary hormones (including follicle stimulating and luteinizing hormones) and ovarian hormones (including estradiol and progesterone). Menstrual bleeding is induced by a drop in progesterone and estradiol concentrations triggered by the absence of fertilization. It is followed by the proliferative phase, characterized by an increase in the production of estradiol by mature follicles in the ovary. The estradiol concentration peaks one to two days before ovulation. Subsequently, the secretory phase begins, characterized by increased production of progesterone (5). Hormone production has been shown to induce compositional changes in the vaginal microbiota in women. At puberty, increased levels of estradiol dramatically alter the vaginal microbiota, at which time it shifts from a diverse microbiota, rich in anaerobic bacteria, to a *Lactobacillus species* (*spp.)* dominant microbiota (6, 7). Exogeneous hormone treatments (injectable progestin, combined oral contraceptives…) also modified bacterial diversity (8–10). In addition, the composition of the vaginal microbiota has also been shown to influence local immune responses. Indeed, a *Lactobacillus* spp. dominated microbiota (Community state type (CST) I, II, V) induces low inflammation and a lower susceptibility to STI acquisition (11, 12). On the contrary, a diverse microbiota composed of anaerobic bacteria, such as *Prevotella, Dialister, Atopium, Gardnerella, Megasphaera, Peptoniphilus, Sneathia*, and *Mobiluncus*, with a low abundance of *Lactobacillus* spp. (CST-IV) has been linked to increased local inflammation. This clinical condition is called bacterial vaginosis (BV). This type of inflammation is mediated by increased expression of pro-inflammatory cytokines, such as IL-1, IL-8, IL-12, IL-18, TNFα, and IFNγ and a higher frequency of activated CD4^+^ T cells and Th17 cells (13). Very little is known about the relationship between neutrophils and the vaginal microbiota. Neutrophils are essential in antimicrobial immunity but a sustained presence of neutrophils within the vagina may lead to tissue damage and inflammation, as seen in several STIs (14, 15). More information is available on the interaction between the gut microbiota and neutrophils. The gut microbiota is essential for neutrophil production and priming and, in return, neutrophils are essential for containment of the microbiota (16–18). Hensley et al. showed that an altered ratio of *Lactobacillus/Prevotella* is responsible for the increased survival of neutrophils in colorectal biopsies of HIV-1-infected Antiretroviral treatment (ART)-treated patients (19).

Here, we focused on the characterization of FRT inflammation during the menstrual cycle and the relationship with local changes in the microbiota in cynomolgus macaques used as a model for the study of human STI.

The FRT of cynomolgus macaques is similar to that of women in terms of morphology, the endocrine system, and menstrual cycle, making them an appropriate study model to analyze the effect of hormonal variation on immune markers (20–22). Menstrual cycle length in cynomolgus macaques is between 28 and 32 days (proliferative phase 12-14 days, secretory phase 14-16 days, bleeding 1-8 days) (21, 22). We previously showed that they have a highly diverse vaginal microbiota, composed mainly of anaerobic bacteria, such as *Sneathia, Porphyromonas, Prevotella, Fusobacterium, Peptoniphilus, Bacteroides*, and *Dialister* and a few *Lactobacillus* spp. This composition is similar to that of women belonging to CST IV (23). Similar to women, the abundance of certain bacterial genera within the vaginal microbiota of female macaques is influenced by hormonal changes (23). These variations observed in the FRT may influence the level of local inflammation and subsequently, susceptibility to STIs.

We characterized the modifications of FRT inflammation during the menstrual cycle in a longitudinal study of nine female cynomolgus macaques. Inflammation was evaluated by measuring cytokine/chemokine concentrations and characterizing neutrophil subpopulations in parallel with determining the composition of the vaginal microbiota. Studying the interactions between the vaginal microbiota and inflammatory markers during the phases of the menstrual cycle is essential to understanding the impact of these factors on susceptibility to STIs and beyond.

## 2 Materials and Methods

### 2.1 Experimental Design and Sample Collection

**Figure 1:**
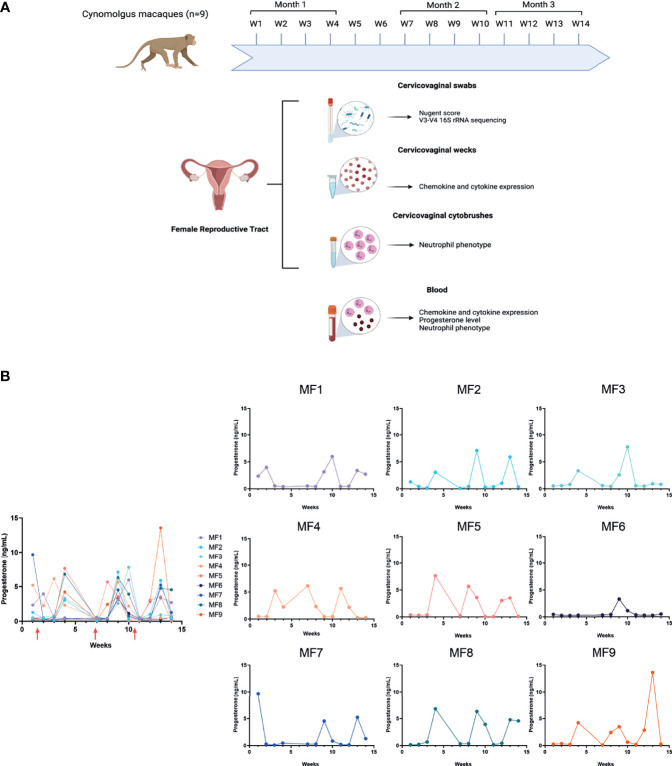
Experimental design and progesterone level. **(A)** Nine female cynomolgus macaques were included in the study. Blood, cervicovaginal fluids (Weck-cel^®^) and swabs, as well as cervicovaginal cells, were collected once a week for three months. Samples were not collected during weeks 5 and 6. Image created in BioRender.com. **(B)** Progesterone concentrations were quantified in plasma once a week for all individuals. The graphical representation of progesterone concentration including all animals was obtained using interpolated and aligned data (left). On the contrary, the graphical representation of progesterone concentration for each individual was obtained using the raw data (right). Red arrows represent menstruation.

Nine female cynomolgus macaques were studied for three months. Their age, weight, and haplotype are summarized in **Table 1**. Sample collection was performed once a week for 12 weeks, representing approximately three menstrual cycles. The experimental design is summarized in **Figure 1A**. The order of sampling was as follows: Weck-Cel Spear during blood withdrawal, cervicovaginal swabs, and cervicovaginal cytobrushes. Cervicovaginal fluids were collected with a Weck-Cel Spear (Medtronic) placed in the vaginal vault for 2 min. Secretions were recovered from the spears by adding 600 µL elution buffer (PBS, 0.25 M NaCl, and protease inhibitor mix; Merck Millipore, Fontenay-sous-Bois, France) and centrifuged at 13,000 x g for 20 min. Secretions were then aliquoted and stored at -80°C before cytokine/chemokine quantification. Blood was collected and used for complete blood counts (CBCs) and the plasma collected, aliquoted, and stored at -80°C for cytokine and progesterone quantification. Vaginal samples for microbiota analysis were collected using nylon flocked swabs with 1mL of liquid amies (ESwabR1, Copan Diagnostics Inc., Murrieta, CA, USA) inserted into the vaginal vault and turned 4 to 5 times before storing in liquid amies. Swabs were then aliquoted and either used for calculation of the Nugent score or stored frozen at -80°C until DNA extraction. Room control samples (air swabs) were performed at the last time point. Cervicovaginal cells were collected using two successive cytobrushes (VWR; Belgium) inserted into the vaginal cavity and turned 4 to 5 times. After collection, the cytobrushes were placed in a 15-mL tube containing 5 mL RPMI with 10% fetal calf serum (FCS) (PAA The cell culture company, ref A15-102, lot: A10210-2737) and 5% penicillin/streptomycin/neomycin (PSN). The samples were kept on ice before processing.

**Table 1:** Information on the nine female cynomolgus macaques included in the study.

Animal	Date of birth	Age (years)	Weight (mean kg ± SD)	Haplotype
MF1	2011-11-24	8.6	6.62 ± 0.06	H6
				H6
MF2	2011-12-27	8.5	4.62 ± 0.08	H5
				H3
MF3	2011-12-26	8.5	3.80 ± 0.12	Rec H2H5
				Rec H3H1
MF4	2010-11-05	9.6	5.42 ± 0.05	H1
				H1
MF5	2010-11-16	9.6	4.05 ± 0.10	H2
				H1
MF6	2012-02-05	8.3	3.76 ± 0.14	H1
				Rec-H1H5H3
MF7	2012-12-02	7.6	6.49 ± 0.09	H3
				H1
MF8	2013-01-19	7.4	5.29 ± 0.12	Rec H6-H11
				Rec H2-H6
MF9	2013-02-05	7.4	5.90 ± 0.07	Rec H3-H2
				Rec H5-H2

### 2.2 Progesterone Quantification

The progesterone level was determined in peripheral blood plasma samples each week by ELISA (IBL international; Germany) according to the manufacturer’s instructions (**Figure 1B**). Progesterone groups were determined as follows: high progesterone (>1.4 ng/mL), low progesterone (<1.4ng/mL), and menstruation (bleeding and/or low progesterone level after a peak of progesterone). We estimated the displacement between the menstrual cycle of the animals (through progesterone levels) using the popular methodology for time series alignment: cross-correlation maximum. Computations were carried out using the Python package SciPy (24). The optimal lag is derived as the one that maximizes the cross-correlation, which is a similarity measure between time-series as a function of the lag.

### 2.3 Cytokine and Chemokine Quantification

Pro- and anti-inflammatory cytokines, as well as chemokines, were quantified in cervicovaginal fluids and plasma using a 23plex assay for the detection of G-CSF, GM-CSF, IFNγ, IL-1β, IL-1RA, IL-2, IL-4, IL-5, IL-6, IL-8, IL-10, IL-12/23(p40), IL-13, IL-15, IL-17A, CCL2, CCL3, CCL4, sCD40L, TGFα, TNFα, VEGF, and IL-18 (NHP cytokine magnetic bead panel kit; Merck Millipore; Germany), according to the manufacturer’s instructions.

### 2.4 Cytospin and Blood Smears

Cytospin was performed at each timepoint on a cervicovaginal cell suspension. Briefly, between 10^4^ and 10^5^ cells were seeded and centrifuged at 72 x g for 1 min. Whole blood smears were also performed for each timepoint. Dry cytospins and whole blood smears were then fixed with Labofix Q path (VWR Avantor; Belgium) and May-Grunwald Giemsa staining performed (RAL diagnostics; France). Slides were observed using an Eclipse 80i microscope (NIKON). Images were acquired with a 60X objective using a high-definition cooled color digital camera (DXM1200C; NIKON).

### 2.5 Neutrophil Phenotyping

Neutrophil populations were analyzed in whole blood and cervicovaginal cells. Cervicovaginal cells were filtered using a 35 µm filter (Corning Falcon; USA). Then, cervicovaginal cells and whole blood were incubated with the antibodies listed in **Table 2**, washed, and fixed with FACS lysing buffer (BD, Biosciences) or BD cell Fix solution (BD, Biosciences). A 14-color panel containing neutrophil maturation and activation surface markers was used. Phenotyping was performed on a Fortessa instrument (BD, Biosciences) using DIVA (BD) and FlowJo (Tristar, USA) software. The gating strategy for cervicovaginal cytobrushes is described in **Supplementary Figure 1**.

**Table 2:** Antibodies used to characterize the neutrophil subpopulations.

Antibody	Clones	Label	Vol per test (~10^6^ cells) or concentration	Reference	Manufacturer
**Bluevid**		BUV736	1X	L23105	Lifetechnologies
**CD64**	10.1	BUV737	3 μL	612776	BD
**CD11b**	REA713	FITC	2 μL	130-110-552	Milteny
**CD45**	REA599	Viogreen	2 μL	130-177-193	Milteny
**CD3**	SP34.2	BV650	2 μL	563916	BD
**CD8**	RPAT8	BV650	2 μL	563821	BD
**CD20**	2H7	BV650	2 μL	563780	BD
**CD123**	7G3	BV650	5 μL	563405	BD
**CD62L**	SK11	BV711	2 μL	565040	BD
**CD14**	REA599	Vioblue	2 μL	130-110-524	Milteny
**CD10**	HI10a	PercP-Cy5,5	5 μL	312216	Biolegend
**CDw125**	REA705	PE	2.5 μL	130-110-544	Milteny
**PD-L1**	29E.2A3	PE-Dazzle594	3 μL	329732	Biolegend
**CD101**	REA954	PE-Vio770	2 μL	130-115-832	Milteny
**CD32a**	IV.3	AF647	0.5 mg/mL	60012	Stemcell
**HLA-DR**	L234	AF700	1 μL	307626	Biolegend
**CD66**	TET2	APC-Vio770	2 μL	130-119-847	Milteny

### 2.6 Nugent Score Determination

Vaginal fluids obtained by swabbing were spread on a slide and fixed with heat (50°C) and 100% ethanol before Gram staining (RAL diagnostics; France). Slides were then observed with a 100X oil immersion lens using an Axioplan 2 microscope (Zeiss). Gram-positive rod-shaped bacteria, as well as non-rod-shaped bacteria, were counted in 10 random fields for each slide. The Nugent score was determined by adding the score of non-rod- and rod-shaped bacteria (**Table 3**). In women, a score from 7 to 10 indicates bacterial vaginosis, from 4 to 6 intermediate flora, and 0 to 3 normal flora (25).

**Table 3:** Nugent score determination in women.

Score	*Lactobacillus* (number of bacteria/field)	Anaerobic bacteria (number of bacteria/field)	*Mobiluncus* (number of bacteria/field)
0	>30	0	0
1	5 to 30	<1	<1 to 4
2	1 to 4	1 to 4	5 to > 30
3	<1	5 to 30	
4	0	>30	

### 2.7 DNA Extraction and 16S rRNA Gene Sequencing

DNA from vaginal fluids was extracted using the PowerFecal DNA Pro isolation kit (Qiagen; Germany) following the manufacturer’s instructions. DNA was quantified on a Qubit 4 fluorimeter using the high sensitivity DNA kit (Life Technologies; USA). PCR and sequencing of the V3-V4 region of the 16S rRNA gene was performed at the @BRIDGe platform (GABI, INRA, AgroParisTech, Paris-Saclay University). The V3-V4 hyper-variable regions of the 16S rRNA gene were amplified from the DNA extracts during the first PCR step using the universal primers 5’ CTTTCCCTACACGACGCTCTTCCGATCTACGGRAGGCAGCAG and 5’ GGAGTTCAGACGTGTGCTCTTCCGATCTTACCAGGGTATCTAATCCT, which are fusion primers (26). The PCR reaction was carried out using a T100 thermal cycler (Biorad, USA) as follows: an initial denaturation step (94°C 10 min) was followed by 30 cycles of amplification (94°C for 1 min, 68°C for 1 min, and 72°C for 1 min) and a final elongation step at 72°C for 10 min. Sample multiplexing was performed by adding tailor-made 6-bp unique indexes during the second PCR step at the same time as the second part of the P5/P7 adapters to obtain primers 5’ AATGATACGGCGACCACCGAGATCTACACT-CTTTCCCTACACGAC and reverse primer 5’ CAAGCAGAAGACGGCATACGAGAT-NNNNNN-GTGACT-GGAGTTCAGACGTGT. The PCR reaction was carried out using a T100 thermal cycler with an initial denaturation step (94°C for 10 min), 12 cycles of amplification (94°C for 1 min, 65°C for 1 min and 72°C for 1 min), and a final elongation step at 72°C for 10 min. All libraries were pooled using equal amounts to generate an equivalent number of raw reads for each library. The pool, at a final concentration of 5 to 20 nM, was used for sequencing. The PhiX Control v3 (Illumina, USA) was added to the pool at 15% of the final concentration, as described in the Illumina procedure. Then, 600 μl of this pool and PhiX mixture was loaded onto an Illumina MiSeq cartridge according to the manufacturer’s instructions using the MiSeq Reagent Kit v3. FastQ files were generated at the end of the run (MiSeq Reporter software, Illumina, USA) to perform the quality control. The quality of the run was checked internally using the PhiX Control and then each paired-end sequence was assigned to its sample using the multiplexing index.

### 2.8 Sequencing Data Processing and Taxonomic Assignation

Illumina sequences were processed using the FROGS pipeline (Find Rapidly OTU with Galaxy Solution) (27). Bacterial 16S rRNA paired-end reads were merged with a 0.1 maximum rate of mismatch in the overlap region using Vsearch (28). Each of the samples was from a unique timepoint. Thus, after dereplication, the clustering step was run with an aggregation distance equal to 1 (maximum number of differences between each of our sequences) and denoising was not required. Chimeras were also removed using Vsearch. More than 93% of the total sequence abundance was retained. Sequences were then filtered to keep at least 0.0005% of all sequences and the phiX databank was equally applied to eliminate Illumina contaminants. Finally, taxonomic affiliation was performed using the SILVA 138 pintail 100 database. Alpha diversity was calculated using the Shannon diversity index. Beta diversity was calculated using the weighted UniFrac distance method.

### 2.9 Statistical Analysis

Heatmaps representing fold changes of cytokine concentrations in cervicovaginal fluids or peripheral blood plasma were obtained using Tableau software (Seattle, USA). Ordinary one-way ANOVA was used to compare fold changes of cytokine concentrations to the mean total fold change as a reference. GraphPad prism software version 9 for windows (GraphPad Software, La Jolla California USA, (http://www.graphpad.com) was used for graphical representation of the vaginal microbiota composition (pie-charts), cytokine concentrations, percentage of neutrophil subpopulations, and bacterial abundance according to progesterone level. Significant differences between groups were confirmed using either a paired T-test or Kruskal-Wallis test with p values adjusted using Dunn’s test. The MetagenomeSeq fitZIG algorithm (29) was used to determine differentially abundant bacteria according to the menstrual phase. Significant differentially abundant cytokines, neutrophils, and microbiome data were merged into one global heatmap using R (version 4.1.0) with the pheatmap package (version 1.0.12). Data were normalized and the fold change calculated using the mean value for the variable in all samples as the reference. A log10 transformation was applied directly to the fold change to manage extreme values. Samples were clustered using a hierarchical clustering approach available in the pheatmap function. Annotation colors were added to visually assess whether the samples clustered by animal or by progesterone level. Finally, absent measurements in the data were flagged as non-determined (ND) and values with an abundance of zero were colored in grey.

### 2.10 Ethical Approval

Nine sexually mature adult female cynomolgus macaques (*Macaca fascicularis*), aged 7 to 9 years and originating from Mauritian AAALAC certified breeding centers, were included in this study. All animals were housed in the Infectious Disease Models and Innovative Therapies facilities (IDMIT) at the Commissariat à l’énergie atomique et aux énergies alternatives (CEA, Fontenay-aux-roses) under BSL-2 containment (Animal facility authorization #D92-032-02, Préfecture des Hauts de Seine, France) and in compliance with European Directive 2010/63/EU, French regulations, and the Standards for the Humane Care and Use of Laboratory Animals of the Office for Laboratory Animal Welfare (OLAW, assurance number #A5826-01, US). This study was approved and accredited by the institutional ethics committee “Comité d’Ethique en Expérimentation Animale du Commissariat à l’Energie Atomique et aux Energies Alternatives” (CEtEA #44) under statement number A18-083. The study was authorized by the “Research, Innovation and Education Ministry” under registration number APAFIS#20692-2019051709424034v1. The nine animals were housed in two different rooms in social groups of 5/6 animals under controlled conditions of humidity, temperature, and light (12 h light/dark cycles). The animals were fed once or twice a day with commercial monkey chow and fruit. Water was available ad libitum. They were provided with environmental enrichment, including toys and novel food under the supervision of the CEA Animal Welfare Officer.

## 3 Results

### 3.1 Cervicovaginal Cytokines Vary According to Hormonal Phase

We monitored local and systemic inflammation by measuring cytokine and chemokine levels in cervicovaginal fluids and peripheral blood plasma using a multiplex technique.

#### 3.1.1 Peripheral Blood Samples

**Figure 2:**
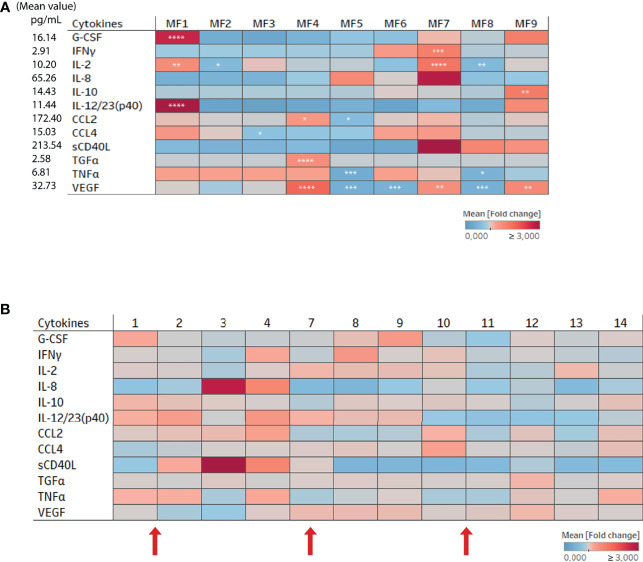
Cytokine and chemokine expression in the plasma of female cynomolgus macaques (n = 9). **(A)** Heatmap representing the mean fold change in the expression of cytokines and chemokines in the plasma of each animal (n = 9). The fold change was calculated based on the mean expression of each cytokine/chemokine for all females and time points. One way ANOVA was performed to compare the total fold change value to those of each animal for each cytokine. Asterisks indicate p values considered to be statistically significant (*p ≤ 0.05, **p ≤ 0.01, ***p ≤ 0.001, ****p ≤ 0.0001). **(B)** Heat map representing the mean fold change in the expression of cytokines and chemokines in the plasma for all animals according to time (n = 9). The fold change was calculated based on the mean expression of each cytokine/chemokine in each female. Numbers 1 to 14 refers to weeks. Red arrows represent menstruation.

Cytokines were quantified in the plasma for each female for all timepoints and the mean fold change calculated based on the total mean value represented for cytokines relevant to the study (**Figure 2A**). The concentration of G-CSF, IFNγ, IL-2, IL-8, IL-10, IL-12/23, CCL2, CCL3, CCL4, sCD40L, TGFα, TNFα, and VEGF varied among the females (**Figure 2A**). For example, monkey MF7 (*Macaca fascicularis* 7) had a higher plasma concentration of IL-2 and IFNγ than the other females, whereas MF1 had a higher plasma concentration of G-CSF, IL-12/23 (p40), and IL-2. The concentration of certain cytokines, such as G-CSF, IL-8, IL12/23 (p40), CCL2, and sCD40L, fluctuated according to time (**Figure 2B**). The samples were then divided into three groups based on progesterone level and clinical observations (high or low progesterone level and menstruation) to further analyze whether the variations were due to the hormonal cycle. There were no significant changes in cytokine concentrations in the plasma due to the hormonal cycle (**Supplementary Figure 2**).

#### 3.1.2 Cervicovaginal Fluids

**Figure 3:**
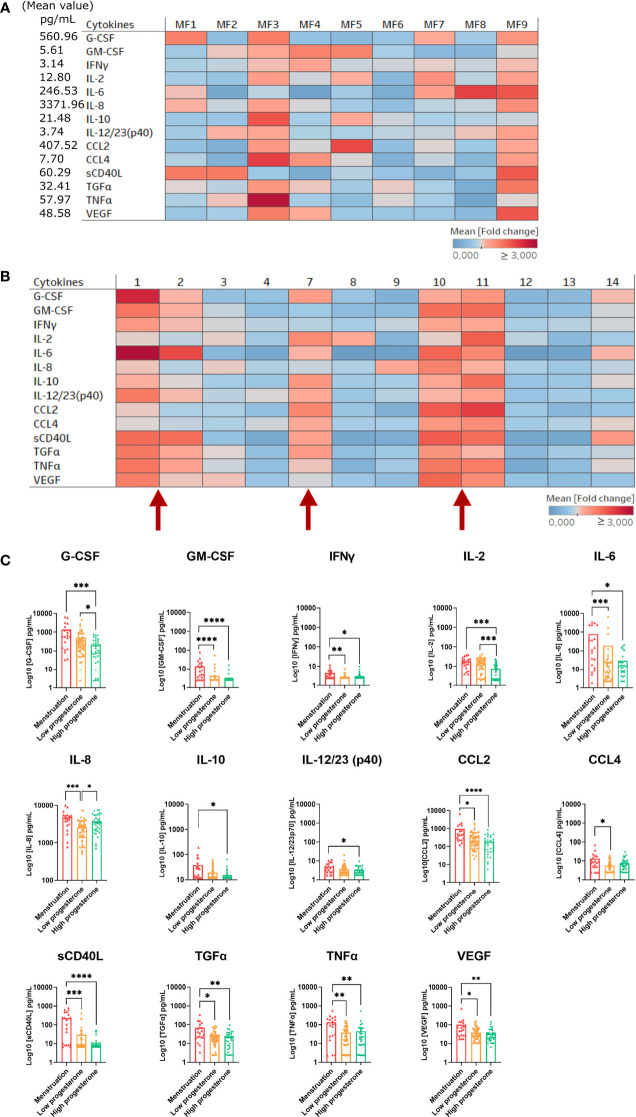
Cytokine and chemokine expression in cervicovaginal fluids of female cynomolgus macaques (n = 9). **(A)** Heatmap representing the mean fold change in the expression of cytokines and chemokines in the cervicovaginal fluids of each animal (n = 9). The fold change was calculated based on the mean expression of each cytokine/chemokine for all females. One way ANOVA was performed to compare the total fold change value to those of each animal for each cytokine. **(B)** Heat map representing the mean fold change in the expression of cytokines and chemokines in the cervicovaginal fluids of all animals (n = 9). The fold change was calculated based on the mean expression of each cytokine/chemokine for each female. Numbers 1 to 14 refers to weeks. Red arrows represent menstruation **(C)** Samples clustered into three groups based on the progesterone level or menstruation and each cytokine/chemokine concentration was plotted. A Kruskal-Wallis test with Dunn’s test to adjust the p value was performed. Asterisks indicate p values considered to be statistically significant (*p ≤ 0.05, **p ≤ 0.01, ***p ≤ 0.001, ****p ≤ 0.0001).

In parallel to peripheral blood plasma, we studied local inflammation in the cervicovaginal fluids. The 23 cytokines were detected in the vaginal fluids of all nine females at varying concentrations. However, only cytokines relevant to this study were displayed on the heatmap representing the mean fold change in expression relative to the total mean value of each cytokine (**Figure 3A**). MF3 expressed a higher level of TNFα and CCL4 than the other females (**Figure 3A**). The heatmap representing the kinetics of cytokine levels in all animals showed a change in cytokine concentrations with time (**Figure 3B**). Interestingly, there was increased production of several cytokines around or during menstruation. All the samples were divided into three groups based on the phase of the menstrual cycle (high or low progesterone level and menstruation) to confirm the impact of the menstrual cycle on cytokine concentration (**Figure 3C**). The analysis showed the concentration of 12 of 23 cytokines/chemokines (CCL2, G-CSF, TNFα, GM-CSF, IFNγ, VEGF, IL-10, IL-12/23, IL-2, TGFα, IL-6, and sCD40L) to be higher in cervicovaginal fluids during menstruation than during the high-progesterone phase. The concentration of CCL4 and IL-8 was higher during menstruation than during the low-progesterone phase. There were also differences between the low- and high-progesterone groups. Indeed G-CSF and IL-2 concentrations were higher in the low-progesterone than high-progesterone group and the opposite was observed for IL-8.

We compared the cytokine concentrations between the blood and cervicovaginal fluids to investigate their similarities and differences. The mean production of several cytokines (IL-1β, IL-1RA, IL-5, IL-6, IL-8, IL-15, IL-18, TGFα, and TNFα), growth factors (GM-CSF, G-CSF), and chemokines (CCL2, CCL3) was higher in the cervicovaginal fluids than peripheral blood plasma for all animals. Only CCL4 was higher in the peripheral blood plasma than cervicovaginal fluids (**Supplementary Figure 3**). Thus, each compartment showed distinct cytokine profiles.

Overall, these results show that the hormonal cycle significantly affects cytokine profiles in cervicovaginal fluids but not the peripheral blood plasma of female cynomolgus macaques.

### 3.2 Neutrophil Subpopulations in Cervicovaginal Cytobrushes Are Strongly Affected by the Hormonal Phase

We collected vaginal cytobrushes and whole blood and stained the cells with specific cell-surface antibodies before flow cytometry analysis to study the presence and evolution of neutrophil subpopulations in the blood and cervicovaginal compartments during the menstrual cycle. Neutrophils were identified as CD45 positive Lineage negative (CD3, CD8, CD20, CD123, CD14, CDw125) and CD66abce positive. CD10 and CD101 were used to determine maturity. CD62L shedding and increased CD11b expression were assessed to determine the priming status of blood neutrophils (30). The panel was completed with CD32a, CD64, PD-L1, and HLA-DR to study activation of the neutrophils in the two compartments.

**Figure 4:**
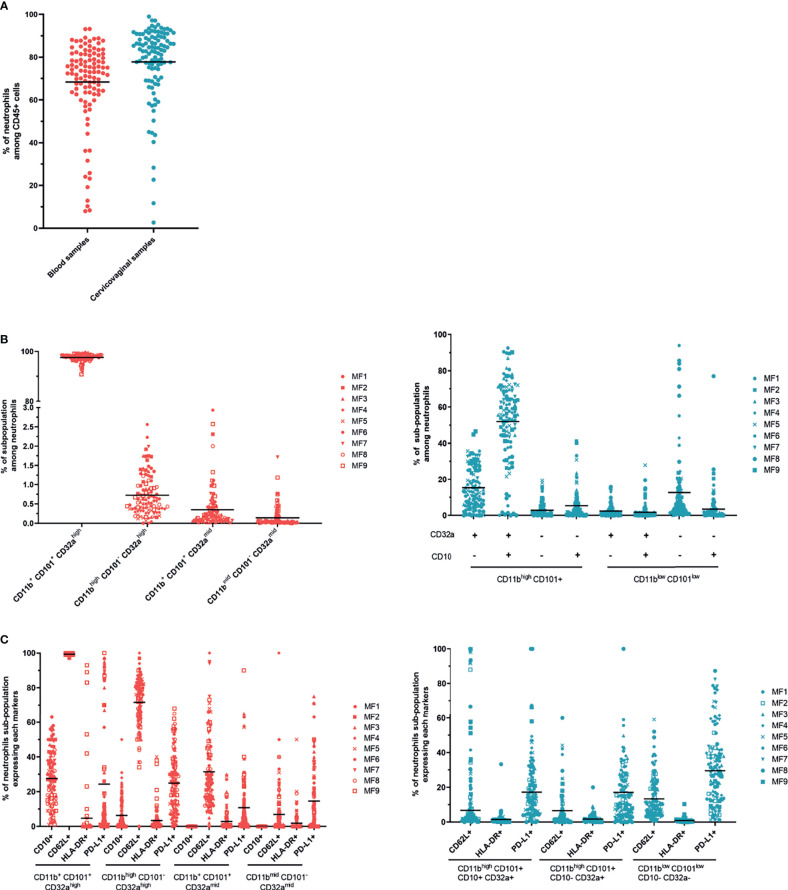
Neutrophil subpopulations in cervicovaginal cytobrushes and blood of female cynomolgus macaques (n = 9). **(A)** Percentage of neutrophils among CD45+ cells in cervicovaginal cytobrushes and blood for all animals. One dot represents one sample of one animal. Blood samples are shown in red and cervicovaginal samples in blue. **(B)** Percentage of neutrophil subpopulations among CD66+ Lin- CD14- CDw125- in blood (left) and cervicovaginal cytobrushes (right) for all females. Each animal is represented by one symbol. **(C)** Percentage of CD62L+, HLA-DR+, and PD-L1+ surface expression for all neutrophil subpopulations in blood (left) and for the main neutrophil subpopulations in cervicovaginal cytobrushes (right).

We first analyzed neutrophil populations in the two compartments independently of their variation with time. The percentage of neutrophils among CD45^+^ cells (Mean ± SD) was quite similar between vaginal cytobrushes (77.8% ± 8.28) and blood (68.3% ± 5.08) in all animals (**Figure 4A**). A punch of vaginal tissue near the cervix was collected and frozen to study the localization of neutrophils within the mucosa. Immunohistochemistry staining was performed using an anti-calprotectin antibody to visualize the neutrophils (**Supplementary Figure 4**), showing the presence of neutrophils underneath the epithelium. This suggests that a portion of the neutrophils collected in cervicovaginal cytobrushes originated from the tissue itself and that another may have come from the lumen. We performed blood smears and cytospins with the cells collected with the cervicovaginal cytobrushes each week to study the morphology of the neutrophils. As seen on an example of a representative individual, most of the neutrophils in the cervicovaginal cytobrushes were actively involved in phagocytosis, in contrast to whole blood neutrophils (**Supplementary Figures 5A, B**). We observed distinct populations in both compartments. Four populations were observed in the blood (**Table 4**): (A) CD11b^+^ CD101^+^ CD32a^high^, (B) CD11b^high^ CD101^–^ CD32a^high^, (C) CD11b^+^ CD101^+^ CD32a^mid^, and (D) CD11b^mid^ CD101^–^ CD32a^mid^ (**Figure 4B**, left). Population A represented 97.54% ± 0.60 (mean from 96.7% to 98.7%) of the CD45^+^ CD66^+^ LinCD3/CD20/CD8a/CDw125/CD14^-^ cells. The three other populations were only weakly present in the blood, with 0.71% ± 0.34 (mean from 0.2% to 1.3%) for population B, 0.35% ± 0.19 for population C (mean from 0.1% to 0.7%), and 0.14% ± 0.08 for population D (mean from 0.06% to 0.3%). No expression of CD10 was observed in population C or D. By contrast, population A (CD11b^+^ CD101^+^ CD32a^high^) expressed a low level of CD10 (mean from 12.5% to 50%), suggesting that a portion of this population was mature (**Figure 4C** left).

**Table 4:** Neutrophil subpopulations characterized in peripheral blood.

CD11b^+^ CD101^+^	CD11b^+^ CD101^+^	CD11b^high^ CD101^-^	CD11b^mid^ CD101^-^
CD32a^high^	CD32a^mid^	CD32a^high^	CD32a^mid^
Population_A	Population_C	Population_B	Population_D

In the blood (**Figure 4C** left), the percentage of cells expressing CD62L varied among the subsets, with 99.3% ± 0.40 (mean from 98.7% to 99.7%) for population A, 71.4% ± 7.14 (mean from 56.1% to 81.7%) for population B, 31.8% ± 13.08 (mean from 17.4% to 57.6%) for population C, and 6.9% ± 4.51 (mean from 2.2% to 13.4%) for population D. We observed a large variation of PD-L1 expression in each population, indeed 23.8% ± 33.41 (mean from 1.8% to 82.2%) for population A, 25.2% ± 12.25 (mean from 4.5% to 42.9%) for population B, 10.8% ± 9.86 (mean from 2.3% to 26.3%) for population C, and 14.5% ± 13.05 (mean from 2.5% to 42.7%) for population D. Expression of HLA-DR and CD64 was low on neutrophils from both the blood and the cervicovaginal cytobrushes at all timepoints. (**Figure 4C** left, Data not shown for CD64).

In conclusion, there was one major population in the blood, characterized as CD11b^+^ CD101^+^ CD32a^high^. This population showed variable expression of CD10, suggesting the presence of both mature and immature subsets. In addition, there was high expression of CD62L and variable expression of PD-L1 in this population. Both mature and immature neutrophils expressed the CD62L marker, which decreases upon priming or tissue migration (30–32). Concerning the immunosuppressive marker PD-L1, its increased expression on blood neutrophils has been detected in patients with sepsis and shown to be associated with reduced phagocytosis and cytokine production (33). We were unable to associate variations in PD-L1 expression with the menstrual cycle or the production of a specific cytokine due to high variability between individuals and timepoints.

Based on the expression of CD11b, CD101, CD10 and CD32, eight populations were determined in cervicovaginal cytobrushes (**Table 5**). However, three main subsets were distinguished: CD11b^high^ CD101^+^ CD10^+^ CD32a^+^ (mean from 18.3% to 67.1%; population 8), CD11b^high^ CD101^+^ CD10^-^ CD32a^+^ (mean from 6.5% to 26.4%; population 6) and CD11b^low^ CD101^low^ CD10^-^ CD32^-^ (mean from 4% to 38%; population 7) (**Figure 4B** right). Based on the expression of CD10, CD101, and CD32a, population 8 can be assigned as a mature/activated subset of neutrophils. The mean CD62L expression in these populations was low in cervicovaginal cytobrushes, with only 17% ± 10.77 (mean from 3.1% to 33.9%) for population 8, 6.48% ± 3.18 (mean from 2.5% to 11.3%) for population 6, and 14.49% ± 4.17 (mean from 7.2% to 19.4%) for population 7. PD-L1 expression was the highest in population 7 (mean from 19.5% to 46.5%), followed by population 8 (mean from 14.2% to 27.1%) and population 6 (mean from 9.6% to 25.6%) (**Figure 4C** right).

**Table 5:** Neutrophil subpopulations characterized in cervicovaginal cytobrushes.

CD11b ^high^ CD101^+^				CD11b ^low^ CD101 ^low^			
CD10^+^		CD10^-^		CD10^+^		CD10^-^	
CD32a^+^	CD32^-^	CD32a^+^	CD32^-^	CD32a^+^	CD32^-^	CD32a^+^	CD32^-^
Population_ 8	Population_ 5	Population_ 6	Population_ 4	Population_ 2	Population_ 1	Population_ 3	Population_ 7

**Figure 5:**
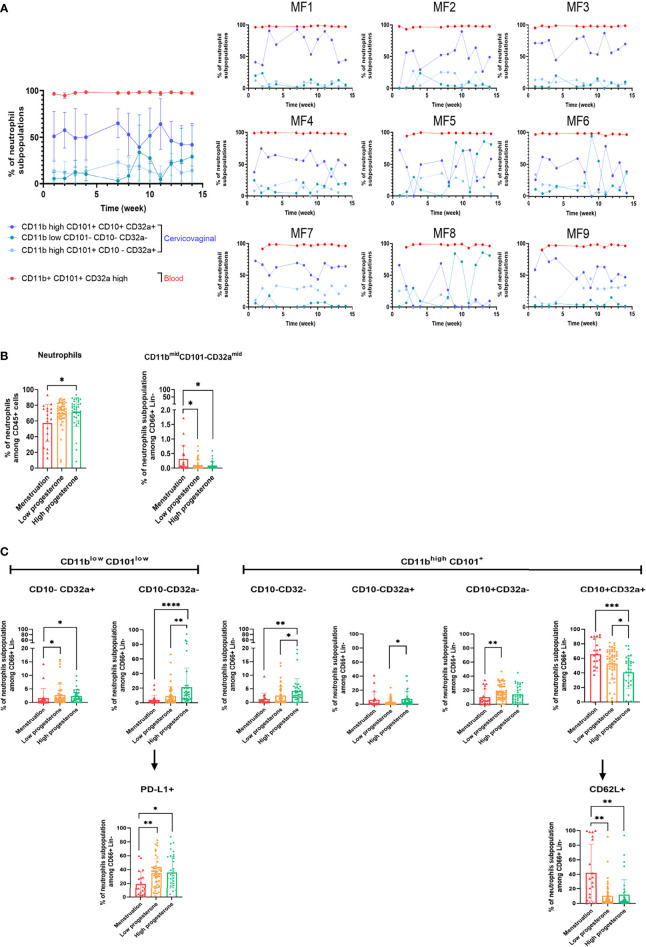
Variation of neutrophil subpopulations in cervicovaginal cytobrushes and blood during the menstrual cycle. **(A)** Main neutrophil subpopulations among CD66+ Lin- cells in both compartments according to the time of collection for all animals (mean of all animals, left) or for each animal (right). Blood **(B)** and cervicovaginal cytobrush **(C)** samples were clustered into three groups based on progesterone levels or menstruation and the percentage of each neutrophil subpopulation was plotted. A Kruskal-Wallis test followed by Dunn’s test to adjust the p value was performed. Asterisks indicate p values considered to be statistically significant (*p ≤ 0.05, **p ≤ 0.01, ***p ≤ 0.001, ****p ≤ 0.0001). Only significant results are presented for **(B)** blood and **(C)** cervicovaginal cytobrushes.

We further studied the kinetics of the main neutrophil populations in the blood and cervicovaginal cytobrushes. The main neutrophil subset in the blood remained stable. However, the percentage of cervicovaginal neutrophil subpopulations fluctuated over time (**Figure 5A**).

We then classified the neutrophil subpopulations in the peripheral blood and cervicovaginal cytobrushes into three groups based on progesterone level and menstruation. The total neutrophil count in peripheral blood was lower during menstruation than the high-progesterone phase. Only the CD11b^mid^ CD101^-^ CD32a^mid^ population varied with progesterone level, peaking during menstruation (**Figure 5B**). On the contrary, we observed greater variation for cervicovaginal neutrophils. Populations 3, 4, and 7 were less abundant during menstruation than during the high-progesterone phase, whereas the abundance of population 8 was significantly higher during menstruation. Moreover, the abundance of populations 4, 6, and 7 were lower in the low-progesterone group than the high-progesterone group, whereas that of population 8 was higher in the low-progesterone than high-progesterone group. Thus, these findings show an increase in neutrophil CD10 expression during menstruation and higher neutrophil CD32a expression during the proliferative than secretory phase. Other markers were also differentially expressed in neutrophils during the menstrual cycle: population 7 expressed lower levels of PD-L1 during menstruation and interestingly, population 8 expressed higher levels of CD62L during menstruation than during the high- and low-progesterone phases (**Figure 5C**).

Overall, these results highlight the presence of neutrophil subpopulations in the cervicovaginal compartment that are affected by the menstrual cycle in cynomolgus macaques. Menstruation induces the accumulation of mature/activated neutrophils that come from the blood based on their high expression of CD62L, which is usually lower in the tissue (32).

### 3.3 The Composition of the Vaginal Microbiota Varies by Hormonal Phase

We have shown that inflammation, translated into cervicovaginal cytokine concentrations and neutrophil subpopulations, varies according to the menstrual cycle. As the vaginal microbiota is involved in the regulation of local inflammation, we characterized the composition of the microbiota in parallel to evaluate its variation during the menstrual cycle.

The composition of the microbiota in each animal was studied using two different approaches: Nugent scoring and V3-V4 16S rRNA sequencing. First, the Nugent score was determined for each female at each timepoint to assess whether the female macaques had a diverse or *Lactobacillus spp* dominant vaginal microbiota. There was very little variation in the Nugent score between most of the females. Indeed, seven had a Nugent score of 7 or 8, depending on the timepoint (**Supplementary Figure 6**). On the contrary, two females (MF6 and MF8) had a variable Nugent score, ranging from 5 to 8. These two females had more Gram-positive bacilli shaped bacteria, which are generally associated with *Lactobacillus* spp. All females showed a diverse vaginal microbiota poor in *Lactobacillus* spp. for most of the timepoints. We then sequenced the V3-V4 region of the 16S rRNA of samples in the study to better characterize the composition of the vaginal microbiota.

**Figure 6:**
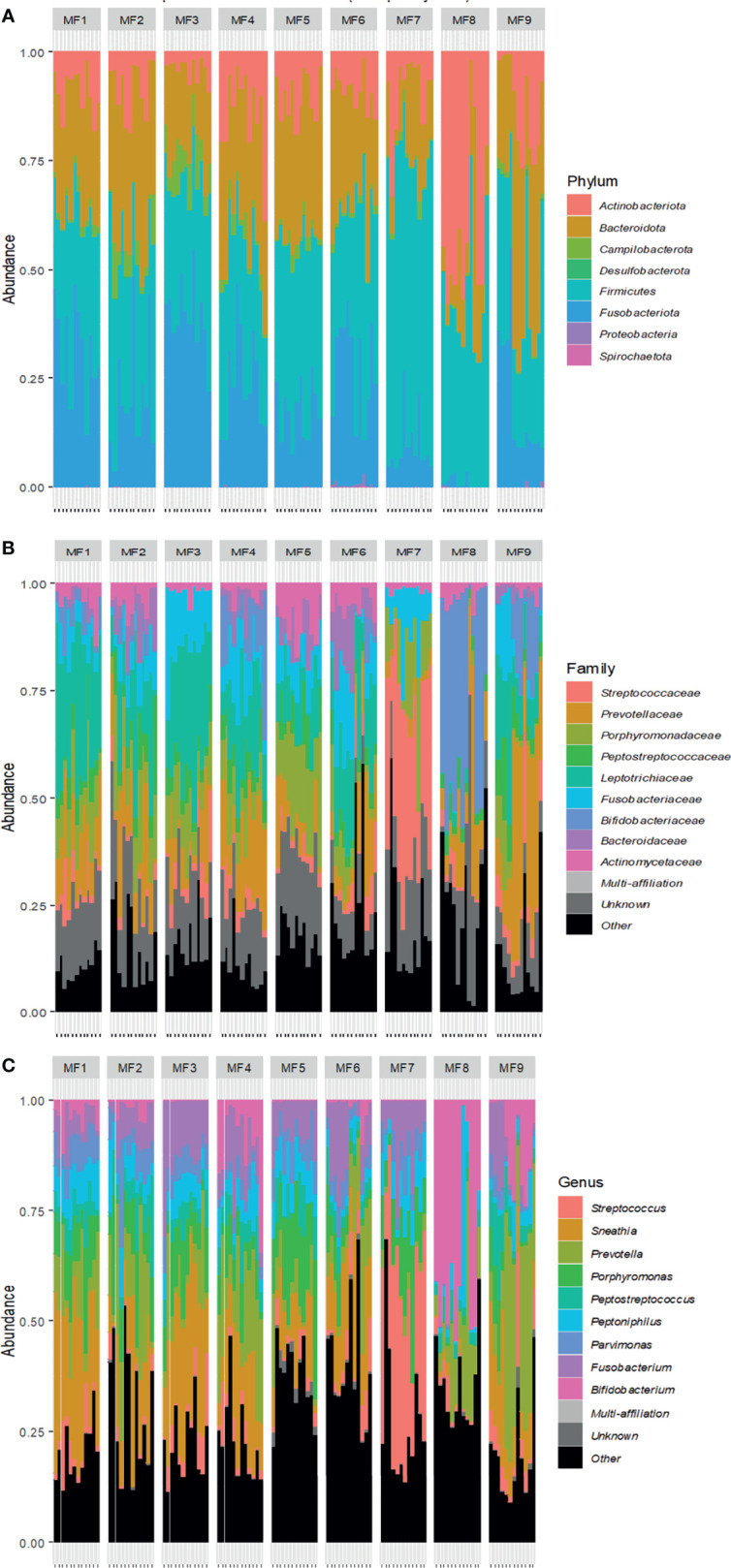
Kinetics of the vaginal microbiota composition of female cynomolgus macaques (n = 9). Relative abundance of bacterial taxa at the phylum level **(A)** in each animal according to time. Relative abundance of the nine most represented genera **(B)** or families **(C)** for all females at all time points and each female according to time. The other families and genera are shown in black (other).

We first identified bacteria present in the vaginal microbiota of each animal. Eight phyla were observed. More than 99% of bacteria in the vaginal microbiota came from five phyla (total mean abundance from highest to lowest): *Firmicutes* (29.7% to 64.4%), *Bacteriodota* (14.7% to 34.9%), *Fusobacteriota* (1.1% to 39.6%), *Actinobacteriota* (3.9% to 40.9%), and *Campilobacterota* (0.24% to 7.3%). The other three phyla (*Desulfobacterota, Proteobacteria* and *Spirochaetota*) represented less than 1% of the total abundance (**Figure 6A** and **Supplementary Figure 7A**). Two females, MF7 and MF8, had a different microbiota composition observed at all taxonomic levels relative to the others (**Figure 6**). Nine predominant families were observed in all females: *Leptotrichiaceae, Prevotellaceae, Porphyromonadaceae, Fusobacteriaceae, Bifidobacteriaceae, Streptococcaceae*, a family from the order *Peptostreptococcales-Tissierellales, Peptostreptococcaceae*, and *Actinomycetaceae* (**Figure 6B**). Nine predominant genera were found in all females, showing variable abundance (mean ± SD) between them: *Sneathia* (13.6% ± 0.12), *Prevotella* (12% ± 0.12), *Porphyromonas* (8.6% ± 0.07), *Fusobacterium* (7.8% ± 0.06), *Streptococcus* (7.2% ± 0.11), *Bifidobacterium* (7% ± 0.13), *Peptoniphilus* (5.9% ± 0.05), *Peptostreptococcus* (4.4% ± 0.04), and *Parvimonas* (4.3% ± 0.03), whereas the mean abundance of the other genera represented 29% (**Figure 6C** and **Supplementary Figure 7B**). *Lactobacillus* spp. were observed in all females but at very low abundance, with MF8 expressing the highest abundance (20.5% ± 0.15) (**Supplementary Figure 7B**). MF8 also had more *Actinobacteriota* (40.87% ± 0.19), related to a higher abundance of *Bifidobacterium* (37.15% ± 0.18). As for MF7, it had more *Firmicutes* (64.38% ± 0.044), translating into a high abundance of *Streptococcus* (35.22% ± 0.12). Moreover, this female also had a very low abundance of *Sneathia* (0.043% ± 0.00) (**Supplementary Figure 7** and **Figures 6A, C**).

**Figure 7:**
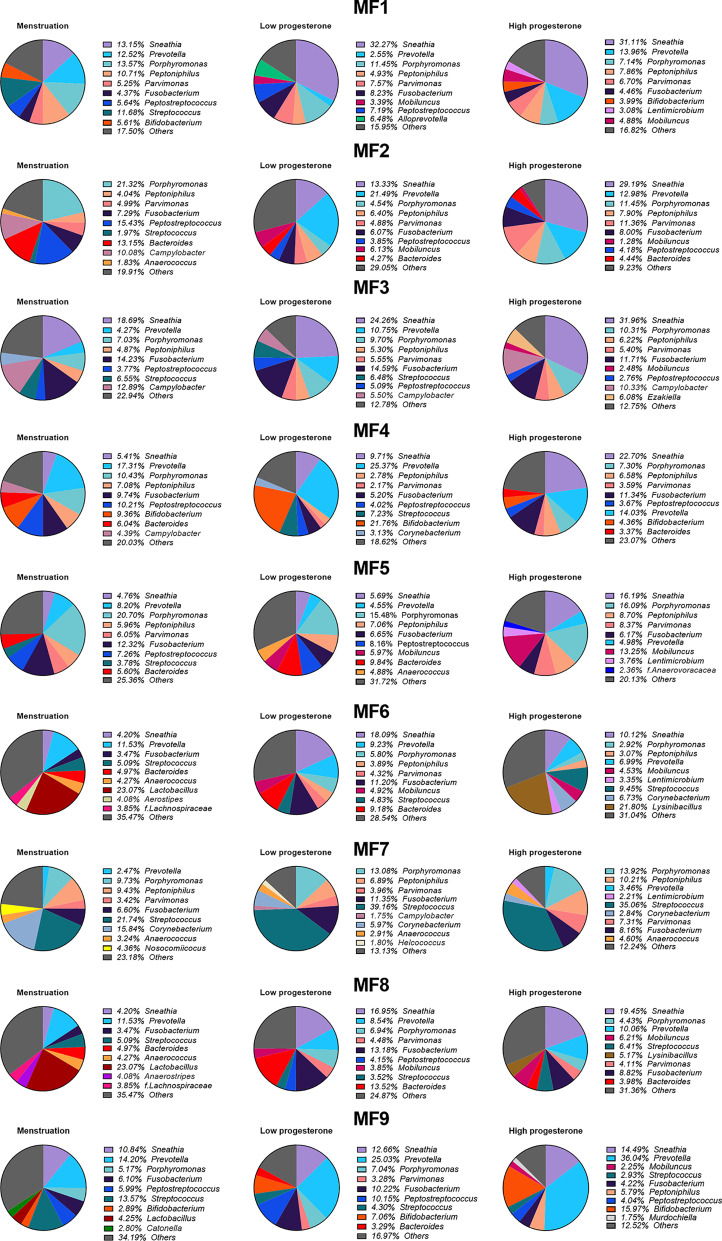
Variation of bacterial taxa during each phase of the menstrual cycle. Percentage of the mean relative abundance of the nine most represented genera in the high-progesterone, low-progesterone, and menstruation groups is represented in a pie chart for each female. Other genera are shown in grey (other).

**Figure 8:**
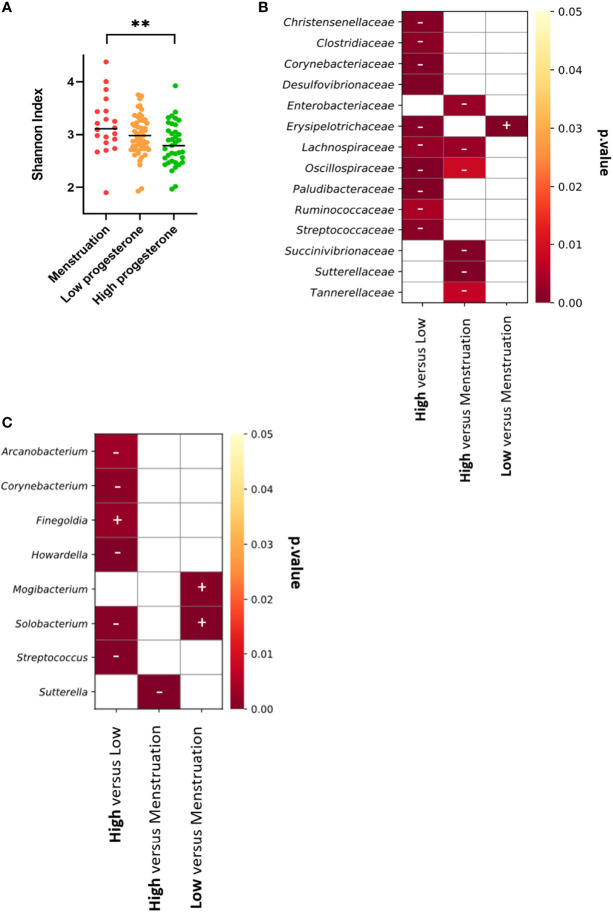
Vaginal microbiota diversity and differentially abundant bacterial taxa in each phase of the menstrual cycle. **(A)** The Shannon index was calculated for each female for the high-progesterone and low-progesterone phases and during menstruation and plotted together. A Kruskal-Wallis test followed by Dunn’s test to adjust the p value was performed (**p ≤ 0.01). Analyses were performed using the FitZIG algorithm at the family **(B)** or genus **(C)** level. Heatmaps representing the p values were generated and plus and minus signs were added to visualize increases or decreases in the abundance of each bacterial taxa. The sign is associated with the group in bold text. As an example: the abundance of *Christensenellaceae* was higher during the high-progesterone phase than the low-progesterone phase.

We represented the top nine genera or families for all or each animal according to the hormonal phase to identify changes in bacterial abundance (**Figure 7** and **Supplementary Figure 8**). Bacterial abundance and composition varied according to the hormonal group for all animals. We assessed whether bacterial diversity was affected by the hormonal phase by calculating the Shannon diversity index according to the high- or low-progesterone or menstruation timepoints. We observed higher bacterial diversity (p value: 0.0065) during menstruation than during the high-progesterone phase (**Figure 8A**). We then performed a statistical analysis of the relative abundance of all families and genera adapted to the high throughput sequencing data for all animals to identify bacterial families and genera that were differentially abundant between progesterone groups (**Figures 8B, C**). This differential analysis identified 14 families that were differentially abundant according to the hormonal group (**Figure 8B**). We generated abundance bar plots to identify increases or decreases based on hormonal group (**Supplementary Figure 9**). The relative abundance of nine families (*Christensellaceae, Clostridiaceae, Corynebacteriaceae, Desulfovibrionaceae, Lachnospiraceae, Oscillospiraceae, Paludibacteraceae, Ruminococcacea*, and *Streptococcaceae)* was lower and one *(Erysipelotrichaceae*) higher in the high-progesterone than low-progesterone group. Six taxa (*Enterobacteriaceae, Lachnospiraceae, Oscillospiracea, Succinivibrionaceae, Sutterellaceae*, and *Tannerellaceae*) were less abundant in the high-progesterone than menstruation group. Finally, *Erysipelotrichaceae* was more abundant in the low-progesterone than menstruation group (**Figure 8B**).

Eight genera (*Arcanobacterium, Corynebacterium, Finegoldia, Howardella, Mogibacterium, Solobacterium, Streptococcus*, and *Sutterella*) were differentially abundant depending on the hormonal phase (**Figure 8C** and **Supplementary Figure 10**). Five genera (*Howardella, Solobacterium, Streptococcus, Sutterella, and Corynebacterium*) are part of the families *Lachnospiraceae, Erysipelotrichaceae, Streptococcacea, Sutterellacea*, and *Corynebacteriaceae* were observed to be differentially abundant according to hormonal group.

In conclusion, the results obtained show that the composition of the vaginal microbiota strongly fluctuated according to hormonal phase in all individuals. The main observations include an enrichment of specific bacterial families/genera at the low-progesterone and menstruation phases relative to the high-progesterone phase.

### 3.4 Menstruation Induces Higher Local Inflammation

**Figure 9:**
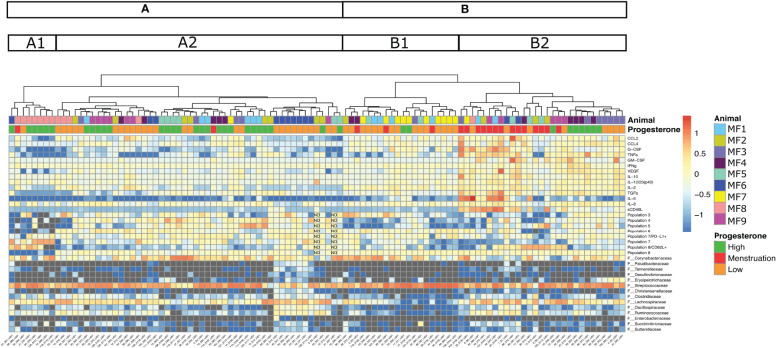
Heatmap representing bacterial taxa (family level), neutrophil subpopulations, and cytokine/chemokine levels that varied during the hormonal cycle. The heatmap represents the log10 fold change for each parameter (cytokines, neutrophil subpopulations, bacterial taxa, indicated on the right). Increased values are shown in red and decreased in blue. Each animal is represented by a color code, as well as the hormonal phases (high progesterone in green, low progesterone in orange, menstruation in red). Hierarchical clustering divided the samples (sample ID in the X-axis) into two large clusters **(A, B)**. Each cluster was then separated into subclusters (A1, A2 and B1, B2).

We wished to obtain a global overview of the association between cytokine expression, neutrophil phenotype and activation, and bacterial abundance in the vagina according to the phase of the menstrual cycle. We thus generated a heatmap supplemented by hierarchical clustering that recapitulated all the variables. This heatmap represents the log10 fold change in cytokine levels, neutrophil populations, and abundance of bacterial taxa at the family level that varied between hormonal phases (**Figure 9**). The hierarchical clustering separated the samples into two large groups (A, B), subdivided into four subgroups (A1, A2, B1, and B2) (**Figure 9**).

The first large group (B) showed low expression of neutrophil subpopulations 3, 4, and 7 and high expression of *Steptococcaceae* in both subgroups. Higher expression of cytokines, such as G-CSF, TNFα, TGFα, IL-6, and sCD40L, as well as a higher presence of neutrophil subpopulation 8 expressing CD62L and *Lachnospiraceae*, was observed in subgroup B2 than in subgroup B1. A lower abundance of the family *Corynebacteriaceae* was also identified in subgroup B2 than in subgroup B1. Subgroup B2 was mainly composed of samples obtained during menstruation (≃48%), followed by low-progesterone (≃27%) and then high-progesterone (≃24%) samples. By contrast, subgroup B1 was dominated by samples obtained during the low-progesterone phase (≃75%), followed by menstruation (≃15%) and high-progesterone (≃10%) samples. Two animals, MF7 (11/12 samples) and MF3 (8/12 samples), were highly represented in group B. In the second group (A), there was lower expression of cytokines and a higher presence of neutrophils than in group B. Overall, we observed that menstruation is associated with an increase in the concentration of cytokines and the accumulation of a specific neutrophil population, characterized as mature (CD101^+^ CD10^+^) and activated (CD32a^+^), that may have originated from the blood (CD62L^+^).

Samples clustered in subgroup A2 showed a small increase in cytokine levels (TGFα and VEGF) and neutrophil subpopulations (3, 4, 5, 6, 7 expressing PD-L1, 7, and 8). However, we observed a decrease in population 8 expressing CD62L relative to subgroup A1, suggesting a lower presence of mature/activated neutrophils originating from the blood. Subgroup A1 showed the low presence of neutrophil subpopulations, such as population 8, and a higher presence of population 7 and higher CD62L expression by population 8, which could be associated with an increased presence of mature neutrophils originating from the blood and weakly activated neutrophils (population 7). Moreover, the abundance of the family *Streptococcaceae* was lower in subgroup A1 than A2. Subgroup A2 was mainly composed of samples collected at the low-progesterone phase (≃54%), followed by high-progesterone (≃44%) and menstruation samples (≃0.2%). In subgroup A1, 75% of the samples were obtained during the high-progesterone phase, followed by low-progesterone and menstruation samples (≃12.5% each). Subgroup A1 was dominated by the samples of one animal, MF8. Indeed, 10 of the 12 samples of MF8 were observed in group A, in which seven were included in subgroup A1, suggesting a strong similarity among the MF8 samples. Concerning MF6, its samples were mainly affiliated with subgroup A2 (9/12 samples) and 7 of 12 were part of a smaller group.

In conclusion, samples collected during menstruation clustered together and were associated with increased inflammation (high cytokine/chemokine production and the accumulation of mature/activated neutrophils that originated from the blood). Moreover, samples retrieve during the high- or low-progesterone phases were distributed throughout all subgroups. Finally, samples of four animals (MF7, MF3, MF8, and MF6) were observed to be highly consistent among them. Samples from two females (MF7, MF3) expressed more cytokines, whereas the other two showed lower cytokine expression (MF8, MF6) but were different in terms of neutrophil subpopulations.

## 4 Discussion

Innate and adaptive immune responses have been described to differ between females and males in animal models and humans, suggesting a strong need to study more female cohorts (34). For example, women infected with HIV-1 exhibit lower plasma viral loads but have a 1.6-fold higher risk for faster progression to the Acquired Immunodeficiency Syndrome (AIDS) (35). Furthermore, vaccine responses are different according to sex; women have higher antibody responses and more local and systemic reactions to bacterial and viral vaccines than men (36, 37). The effect of hormones on immune cell recruitment, activation, and soluble factor concentrations in peripheral blood and cervicovaginal lavages of the FRT has been partially studied at each menstrual phase in women. However, there are technical disparities among the studies, such as the absence of hormone measurements and non-longitudinal analysis.

In this study on female cynomolgus macaques, we investigated variations in the vaginal microbiota, cytokine expression, and neutrophil subpopulations in the vagina and blood in a longitudinal study covering three complete menstrual cycles. We show that cervicovaginal environmental factors, such as cytokine and chemokine concentrations, neutrophil populations, and microbiota vary during the menstrual cycle. We highlight that menstruation induces significant alteration of the environment by increasing local inflammation, with an increase of cytokine/chemokine production (GM-CSF, G-CSF, IFNγ, IL-6, CCL2, sCD40L, TGFα, TNFα, VEGF…) and the accumulation of CD11b^+^ CD101^+^ CD32a^+^ CD62L^+^ neutrophils that may originate in the blood. Menstruations lead to an injured endometrium by inducing the shedding of the upper functional layer of the endometrium. The menstruating endometrium triggers an inflammatory response that is necessary for a rapid tissue repair (38). Therefore, the inflammation, associated with an increased production of cytokines such as CCL2, G-CSF, GM-CSF or IL-6, is able to trigger neutrophils recruitment, which might facilitate tissue repair after menstruation.

At steady state, we show that cytokine concentrations are low in the peripheral blood relative to their expression in cervicovaginal fluids. Analysis of culture supernatants of cells isolated from the human FRT and peripheral blood mononuclear cells (PBMCs) show similar differences in terms of cytokine and chemokine expression between the systemic and local compartments (39). Our findings show variability in terms of cytokine production in both compartments according to the animal and the time of the measurement. Nevertheless, only cervicovaginal cytokine production was influenced by the hormonal phase, characterized by a significant increase in cytokine and chemokine concentrations during menstruation. A comparison of cytokine concentrations between menstrual and peripheral blood of women showed differences similar to our results in macaques (40). Several human cohort-based studies have reported variations in cytokine and chemokine levels according to the hormonal cycle in cervicovaginal fluids, but the results were inconsistent and none of the studies collected samples during menstruation (41–43).

Together with the cytokine profiles, we characterized neutrophil subpopulations in the blood and cervicovaginal cytobrushes in female cynomolgus macaques at different time points. The study of Lemaître et al. showed neutrophils in the blood of cynomolgus macaques to be CD45^+^ CD66^+^ Lin(CD3/CD20/CD8a/CDw125/CD14^-^) and used CD11b/CD32a expression to determine the maturation stage (44). In our study, we added CD10 and CD101 to confirm the maturation status of neutrophils. In female cynomolgus macaques, the percentage of neutrophils among CD45^+^ cells was similar between the peripheral blood and cervicovaginal cytobrushes. Throughout the study, peripheral blood neutrophils had either an immature or mature phenotypes. In contrast, in cervicovaginal cytobrushes, the expression of various markers of neutrophils differed from the one in blood. Indeed, three main populations of cervicovaginal neutrophils were observed, which can be identified, based on the characterization of neutrophils in the blood, as mature/activated (population 8), immature/activated (population 6) and pre-neutrophil like (population 7). Population 7 has a phenotype of pre-neutrophils, a population usually found specifically in the bone marrow (45). May-Grunwald Giemsa staining of neutrophils from the cervicovaginal cytobrushes showed them to be mainly poly-nuclear and no pre-neutrophils, with a round nucleus, were observed (**Supplementary Figure 3**). The abundance of the main neutrophil populations in cervicovaginal cytobrushes varied according to the hormonal phase. This was not the case for blood neutrophils. More precisely, the abundance of the mature/activated subset expressing CD62L in the cervicovaginal compartment increased during menstruation. This could be due to infiltration of the FRT by mature/activated blood neutrophils because CD62L expression on neutrophils decreases after transmigration into the tissue (31). Moreover, an increase in the concentration of CCL2, G-CSF, GM-CSF, and IL-6 was previously reported to be associated with local neutrophil recruitment upon infection (46–49). Overall, distinct subsets of neutrophils were identified in the blood and in the cervicovaginal compartment. The abundance of the main subset of neutrophils in the cervicovaginal compartment varied according to the hormonal phase in contrast to blood neutrophils.

Neutrophil populations in human blood have been extensively described and characterized as CD15^+^ CD66b^+^ CD49d^mid^ CD101^+^ CD10^+^ CD16^+^ CD62L^+^ (45). Moreover, they increase during the secretory phase (high progesterone concentration), as we observed in cynomolgus macaques (50). Thus far, neutrophils have been studied in the human endometrium and were shown to increase early during the menstrual cycle, leading the authors to hypothesize that such an increase is necessary for tissue repair after menstruation (51). We can assume that the same phenomenon occurs in cynomolgus macaque. Our study is the first to analyze the evolution of innate immune markers, including cervicovaginal neutrophils and cytokine profiles, together with the composition of the vaginal microbiota, according to the phases of the menstrual cycle.

The vaginal microbiota has been described to be closely involved in local immune regulation (13). We thus longitudinally monitored the composition of the vaginal microbiota in parallel with immune marker analysis. We show a predominance of four phyla: *Firmicutes, Bacteroidota, Fusobacteriota*, and *Actinobacteriota*. The predominant genera were *Sneathia, Prevotella, Porphyromonas, Fusobacterium, Streptococcus, Bifidobacterium, Peptoniphilus, Peptostreptococcus*, and *Parvimonas*, in accordance with our previously published study on the characterization of the vaginal and rectal microbiota of female cynomolgus macaques (23). We show that the composition of the vaginal microbiota is subject to hormonal changes. In our previously published study, we did not consider menstruation. Here, we describe that the diversity of the vaginal microbiota increases during menstruation. Increased bacterial diversity during menstruation has also been observed in women, demonstrated by a trend towards the increased abundance of several bacterial taxa, including *Finegoldia, Streptococcus*, and *Peptostreptococcus* (2, 52). The abundance of none of these genera significantly increased during menstruation in cynomolgus macaques. These differences could be because most of samples in the study of Song et al. came from women with a *Lactobacillus* spp. dominant microbiota, whereas macaques have a diverse vaginal microbiota similar to dysbiosis in women. During menstruation, six families of bacteria are increased in cynomolgus macaques, including *Lachnospiraceae* and *Succinivibrionaceae*. The modification of the environment associated with the presence of menstrual blood might be responsible for those changes. For instance, iron increase might facilitate the presence of other bacterial families. In fact, in women, iron increase during menstruation is associated with the growth of *G. vaginalis*, a BV associated bacteria (53).

Finally, we analyzed the relationships between immune factors and bacterial taxa that showed differential abundance according to the hormonal phase. The analysis resulted in the clear clustering of samples collected during menstruation. Such clustering suggests increased inflammation (i.e., an increase in cytokine production and mature/activated neutrophils) during menstruation that is not observed during the other hormonal phases. On the contrary, high-progesterone (secretory phase) and low-progesterone (proliferative phase) samples mostly exhibited lower cytokine expression (mainly CCL2, CCL4, IL-6, and sCD40L) and a higher number of less mature and activated neutrophil populations than during menstruation. There were few differences between samples collected during the high- and low-progesterone phases. The analysis also showed animal-specific clusters for four females, whereas the others had a more variable distribution. This observation demonstrates not only strong variability between animals but also within each animal, confirming a specific individual mucosal environment. This is likely why we were unable to associate a group of bacteria or a specific genus to a cytokine profile and/or a neutrophil phenotype. Modification of the vaginal microbiota composition by antibiotic treatment and/or probiotic inoculation could be helpful for this purpose. The determination of such associations would be useful for the development of therapeutic approaches aiming to modify the vaginal microbiota to reduce local inflammation.

In terms of cytokine profiles and neutrophil subpopulations, both the peripheral blood and cervicovaginal compartments were significantly different, highlighting that the blood compartment does not recapitulate what happens in the mucosal compartment. It is thus necessary to investigate the mucosal compartment and not only the systemic one. Our study was performed at steady state, but infection or vaccination could exacerbate the differences between compartments. Moreover, our results suggest that immune markers and the vaginal microbiota fluctuate within the FRT at the basal state. Such fluctuations are mainly due to the hormonal cycle and must be considered when studying STI infections or mucosal vaccine responses in the mucosa. Indeed, an increase in cytokine expression could be believed to be associated with a vaccine response or immune response against a pathogen, whereas it may, in fact, be due to a physiological change, for example, during menstruation. The impact of hormones has been described in the acquisition of STIs. Indeed, estradiol has been shown to have a positive effect on the mucosal environment by increasing the thickness of the vaginal epithelium and favoring the growth of *Lactobacillus* (2, 7). On the contrary, injectable progestin such as depot medroxyprogesterone acetate (DMPA or MPA) favors an increased bacterial diversity and a decreased of *Lactobacillus spp* likely through a reduction of endogenous estrogen, glycogen and α amylase (8, 10). In addition, progesterone reduces the thickness of the vaginal epithelium in rhesus macaques and increases the incidence of the Simian Immunodeficiency Virus (SIV) (54). These findings highlight the need for extensive characterization of the hormonal impact on the mucosa of the FRT by an appropriate sampling schedule, including several time points. Our data highlight the importance of longitudinal studies for the analysis of immune markers and/or the composition of the vaginal microbiota in humans and animal models. The sampling has to be repeated to account for potential hormonal cycle effects. Otherwise, the results obtained may be biased.

This longitudinal study provides insights into how the phases of the menstrual cycle affect the composition of the vaginal microbiota, as well as that of immune markers, such as cytokine profiles and neutrophil subpopulations. It consequently highlights the difficulties associated with studying the local environment in the FRT and the need for more women-based cohort studies with an appropriate sampling schedule. This complex hormonal regulation needs to be further addressed by including other factors involved in mucosal immunity, such as IgA/IgG quantification and that of immune cells, which have been described to vary in the human endometrium according to the hormonal phase (T and B lymphocytes, Natural Killer and myeloid cells) (55). Further studies should quantify other hormones such as estradiol or FSH in addition to progesterone to better evaluate the hormonal cycle. A better understanding of how hormones regulate the microbiota and immune factors within the FRT is essential to better characterize their impact on the control of acquisition of STIs and mucosal vaccine responses.

## Data Availability Statement

The datasets presented in this study can be found in online repositories. The names of the repository/repositories and accession number(s) can be found below: https://www.ncbi.nlm.nih.gov/, Bioproject ID PRJNA768787. Other datasets generated or analyzed during the current study are available from the corresponding author on reasonable request.

## Ethics Statement

The animal study was reviewed and approved by Comité d’Ethique en expérimentation animale du commissariat à l’énergie atomique et aux énergies alternatives (CEtEA#44) under statement number (A18-083) and was authorized by the “Research, Innovation and Education Ministry” under registration number APAFIS#20692-2019051709424034v1.

## Author Contributions

The study was conceived and designed by CA, LR, NN, RM, JL, RLG, M-TN, and EM. The acquisition of the data was performed by CA, LR, CC, and M-TN. Analysis and interpretation of the data was done by CA, LR, NN, Ld’A, EmG, ErG, M-TN, and EM. CA and EM drafted the manuscript. Critical revisions were performed by CA, LR, NN, RM, JL, RLG, M-TN, and EM. The authors declare that they have no competing interests. All authors contributed to the article and approved the submitted version.

## Funding

The program was funded by the Infectious Disease Models and Innovative Therapies (IDMIT) research infrastructure supported by the “Programme Investissements d’Avenir”, managed by the ANR under reference ANR-11-INBS-0008, as well as Sidaction “financement jeune chercheur” under reference “2019-2-FJC-1234”. The Non-Human Primate study is part of the TracVac project, which received financial support from the European Union’s Horizon 2020 research and innovation program under grant agreement No. 733373.

## Conflict of Interest

Authors NN, Ld'A, EmG and ErG were employed by Life&Soft.

The remaining authors declare that the research was conducted in the absence of any commercial or financial relationships that could be construed as a potential conflict of interest.

## Publisher’s Note

All claims expressed in this article are solely those of the authors and do not necessarily represent those of their affiliated organizations, or those of the publisher, the editors and the reviewers. Any product that may be evaluated in this article, or claim that may be made by its manufacturer, is not guaranteed or endorsed by the publisher.

## Supplementary Material

Supplementary Figure 1 FACS gating stategy for neutrophil phenotyping in blood samples **(A)** and cervicovaginal cytobrushes **(B)** of one representative animal.

Supplementary Figure 2 Cytokine and chemokine expression in the plasma of female cynomolgus macaques according to the phase of the hormonal cycle (n = 9). Samples were clustered into three groups based on progesterone level or menstruation and each cytokine/chemokine concentration (pg/mL) was plotted. A Kruskal-Wallis test followed by Dunn’s test to adjust the p value was performed.

Supplementary Figure 3 Cytokine and chemokine expression in the plasma and cervicovaginal fluids (CVF) of female cynomolgus macaques. The mean concentration of each cytokine/chemokine for each animal in the plasma and CVF were plotted and a paired T-test was performed to compare the two compartments. Asterisks indicate p values considered to be statistically significant (*p ≤ 0.05, **p ≤ 0.01, ***p ≤ 0.001, ****p ≤ 0.0001).

Supplementary Figure 4 Localization of neutrophils in the vaginal mucosa. Immunohistochemistry staining of a slide obtained from a vaginal punch (upper vagina near the cervix) of one female cynomolgus macaque. An anti-calprotectin antibody labelled with FITC (green) was used to stain neutrophils and DAPI to stain the nucleus (blue).

Supplementary Figure 5 Neutrophil morphology in the blood and cervicovaginal cytobrushes. Neutrophil staining by May-Grunwald-Giemsa of **(A)** cervicovaginal cells obtained from cytobrushes and **(B)** blood smears of one representative animal (MF7) for all time points. The red star represents menstruation and the black arrow neutrophils.

Supplementary Figure 6 Nugent scores for each female during the three months follow-up.

Supplementary Figure 7 Vaginal microbiota composition of the female cynomolgus macaques (n = 9). The percentage of the mean relative abundance of phyla **(A)** or the top nine most represented genera **(B)** for all animals (top) or for each female (bottom) are represented in the pie chart.

Supplementary Figure 8 Variation of the abundance of bacterial taxa in each phase of the menstrual cycle. The mean relative abundance of the nine most represented genera (top) and families (below) in the high-progesterone, low-progesterone, and menstruation groups is represented in the pie chart for all females. Other genera are shown in grey (other).

Supplementary Figure 9 Graphical representation of the relative abundance of bacterial taxa according to the hormonal phase at the family level. Only bacterial taxa that were differentially expressed according to the hormonal phase are represented.

Supplementary Figure 10 Graphical representation of the relative abundance of bacterial taxa according to the hormonal phase at the genus level. Only bacterial taxa that were differentially expressed according to the hormonal phase are represented.

## References

[1] Wira CR Rodriguez-Garcia M Patel MV. The Role of Sex Hormones in Immune Protection of the Female Reproductive Tract. Nat Rev Immunol (2015) 15:217–30. doi: 10.1038/nri3819 PMC4716657 25743222

[2] Song SD Acharya KD Zhu JE Deveney CM Walther-Antonio MRS Tetel MJ. Daily Vaginal Microbiota Fluctuations Associated With Natural Hormonal Cycle, Contraceptives, Diet, and Exercise. mSphere (2020) 5(4):e00593-20. doi: 10.1128/mSphere.00593-20 32641429 PMC7343982

[3] Gajer P Brotman RM Bai G Sakamoto J Schütte UME Zhong X. Temporal Dynamics of the Human Vaginal Microbiota. Sci Transl Med (2012) 4:132ra52. doi: 10.1126/scitranslmed.3003605 PMC3722878 22553250

[4] Boily-Larouche G Lajoie J Dufault B Omollo K Cheruiyot J Njoki J. Characterization of the Genital Mucosa Immune Profile to Distinguish Phases of the Menstrual Cycle: Implications for HIV Susceptibility. J Infect Dis (2019) 219:856–66. doi: 10.1093/infdis/jiy585 PMC6386813 30383238

[5] Mihm M Gangooly S Muttukrishna S. The Normal Menstrual Cycle in Women. Anim Reprod Sci (2011) 124:229–36. doi: 10.1016/j.anireprosci.2010.08.030 20869180

[6] Dei M Di Maggio F Di Paolo G Bruni V. Vulvovaginitis in Childhood. Best Pract Res Clin Obstetrics Gynaecol (2010) 24:129–37. doi: 10.1016/j.bpobgyn.2009.09.010 19884044

[7] Mirmonsef P Hotton AL Gilbert D Burgad D Landay A Weber KM. Free Glycogen in Vaginal Fluids is Associated With Lactobacillus Colonization and Low Vaginal pH. PloS One (2014) 9:e102467. doi: 10.1371/journal.pone.0102467 25033265 PMC4102502

[8] Jespers V Kyongo J Joseph S Hardy L Cools P Crucitti T. A Longitudinal Analysis of the Vaginal Microbiota and Vaginal Immune Mediators in Women From Sub-Saharan Africa. Sci Rep (2017) 7:11974. doi: 10.1038/s41598-017-12198-6 28931859 PMC5607244

[9] Brooks JP Edwards DJ Blithe DL Fettweis JM Serrano MG Sheth NU. Effects of Combined Oral Contraceptives, Depot Medroxyprogesterone Acetate, and the Levonorgestrel-Releasing Intrauterine System on the Vaginal Microbiome. Contraception (2017) 95:405–13. doi: 10.1016/j.contraception.2016.11.006 PMC5376524 27913230

[10] Wessels JM Lajoie J Cooper MIJH Omollo K Felker AM Vitali D. Medroxyprogesterone Acetate Alters the Vaginal Microbiota and Microenvironment in a Kenyan Sex Worker Cohort and is Also Associated With Increased Susceptibility to HIV-1 in Humanized Mice. Dis Models Mech (2019) 12(10):dmm039669. doi: 10.1242/dmm.039669 PMC6826019 31537512

[11] Gosmann C Anahtar MN Handley SA Farcasanu M Abu-Ali G Bowman BA. Lactobacillus-Deficient Cervicovaginal Bacterial Communities Are Associated With Increased HIV Acquisition in Young South African Women. Immunity (2017) 46:29–37. doi: 10.1016/j.immuni.2016.12.013 28087240 PMC5270628

[12] Tamarelle J Thiébaut ACM de Barbeyrac B Bébéar C Ravel J Delarocque-Astagneau E. The Vaginal Microbiota and its Association With Human Papillomavirus, Chlamydia Trachomatis, Neisseria Gonorrhoeae and Mycoplasma Genitalium Infections: A Systematic Review and Meta-Analysis. Clin Microbiol Infect (2019) 25:35–47. doi: 10.1016/j.cmi.2018.04.019 29729331 PMC7362580

[13] Anahtar MN Byrne EH Doherty KE Bowman BA Yamamoto HS Soumillon M. Cervicovaginal Bacteria are a Major Modulator of Host Inflammatory Responses in the Female Genital Tract. Immunity (2015) 42:965–76. doi: 10.1016/j.immuni.2015.04.019 PMC4461369 25992865

[14] Stevens JS Criss AK. Pathogenesis of Neisseria Gonorrhoeae in the Female Reproductive Tract: Neutrophilic Host Response, Sustained Infection, and Clinical Sequelae. Curr Opin Hematol (2018) 25:13–21. doi: 10.1097/MOH.0000000000000394 29016383 PMC5753798

[15] Lijek RS Helble JD Olive AJ Seiger KW Starnbach MN. Pathology After Chlamydia Trachomatis Infection is Driven by Nonprotective Immune Cells That are Distinct From Protective Populations. Proc Natl Acad Sci USA (2018) 115:2216–21. doi: 10.1073/pnas.1711356115 PMC5834673 29440378

[16] Deshmukh HS Liu Y Menkiti OR Mei J Dai N O’Leary CE. The Microbiota Regulates Neutrophil Homeostasis and Host Resistance to Escherichia Coli K1 Sepsis in Neonatal Mice. Nat Med (2014) 20:524–30. doi: 10.1038/nm.3542 PMC4016187 24747744

[17] Zhang D Frenette PS. Cross Talk Between Neutrophils and the Microbiota. Blood (2019) 133:2168–77. doi: 10.1182/blood-2018-11-844555 PMC6524562 30898860

[18] Zhang D Chen G Manwani D Mortha A Xu C Faith JJ. Neutrophil Ageing Is Regulated by the Microbiome. Nature (2015) 525:528–32. doi: 10.1038/nature15367 PMC4712631 26374999

[19] Hensley-McBain T Wu MC Manuzak JA Cheu RK Gustin A Driscoll CB. Increased Mucosal Neutrophil Survival is Associated With Altered Microbiota in HIV Infection. PloS Pathog (2019) 15:e1007672. doi: 10.1371/journal.ppat.1007672 30973942 PMC6459500

[20] Van Esch E Cline JM Buse E Wood CE de Rijk EPCT Weinbauer GF. Summary Comparison of Female Reproductive System in Human and the Cynomolgus Monkey (Macaca Fascicularis). Toxicol Pathol (2008) 36:171S–2S. doi: 10.1177/0192623308327415

[21] Weinbauer GF Niehoff M Niehaus M Srivastav S Fuchs A Van Esch E. Physiology and Endocrinology of the Ovarian Cycle in Macaques. Toxicol Pathol (2008) 36:7S–23S. doi: 10.1177/0192623308327412 20852722 PMC2939751

[22] Shaikh AA Naqvi RH Shaikh SA. Concentrations of Oestradiol-17beta and Progesterone in the Peripheral Plasma of the Cynomolgus Monkey (Macaca Fascicularis) in Relation to the Length of the Menstrual Cycle and Its Component Phases. J Endocrinol (1978) 79:1–7. doi: 10.1677/joe.0.0790001 101640

[23] Nugeyre M-T Tchitchek N Adapen C Cannou C Contreras V Benjelloun F. Dynamics of Vaginal and Rectal Microbiota Over Several Menstrual Cycles in Female Cynomolgus Macaques. Front Cell Infect Microbiol (2019) 9:188. doi: 10.3389/fcimb.2019.00188 31249812 PMC6582644

[24] Virtanen P Gommers R Oliphant TE Haberland M Reddy T Cournapeau D. SciPy 1.0: Fundamental Algorithms for Scientific Computing in Python. Nat Methods (2020) 17:261–72. doi: 10.1038/s41592-019-0686-2 PMC7056644 32015543

[25] Nugent RP Krohn MA Hillier SL. Reliability of Diagnosing Bacterial Vaginosis Is Improved by a Standardized Method of Gram Stain Interpretation. J Clin Microbiol (1991) 29:297–301. doi: 10.1128/jcm.29.2.297-301.1991 1706728 PMC269757

[26] Nadkarni MA Elizabeth M Jacques NA Hunter N. Determination of Bacterial Load by Real-Time PCR Using a Broad-Range (Universal) Probe and Primers Set. Microbiology (2002) 148:257–66. doi: 10.1099/00221287-148-1-257 11782518

[27] Escudié F Auer L Bernard M Mariadassou M Cauquil L Vidal K. FROGS: Find, Rapidly, OTUs With Galaxy Solution. Bioinformatics (2018) 34:1287–94. doi: 10.1093/bioinformatics/btx791 29228191

[28] Rognes T Flouri T Nichols B Quince C Mahé F. VSEARCH: A Versatile Open Source Tool for Metagenomics. PeerJ (2016) 4:e2584. doi: 10.7717/peerj.2584 27781170 PMC5075697

[29] Paulson JN Stine OC Bravo HC Pop M. Differential Abundance Analysis for Microbial Marker-Gene Surveys. Nat Methods (2013) 10:1200–2. doi: 10.1038/nmeth.2658 PMC4010126 24076764

[30] Condliffe AM Chilvers ER Haslett C Dransfield I. Priming Differentially Regulates Neutrophil Adhesion Molecule Expression/Function. Immunology (1996) 89:105–11. doi: 10.1046/j.1365-2567.1996.d01-711.x PMC1456672 8911147

[31] Ivetic A. A Head-to-Tail View of L-Selectin and Its Impact on Neutrophil Behaviour. Cell Tissue Res (2018) 371:437–53. doi: 10.1007/s00441-017-2774-x PMC5820395 29353325

[32] Lee D Schultz JB Knauf PA King MR. Mechanical Shedding of L-Selectin From the Neutrophil Surface During Rolling on Sialyl Lewis X Under Flow*. J Biol Chem (2007) 282:4812–20. doi: 10.1074/jbc.M609994200 17172469

[33] Patera AC Drewry AM Chang K Beiter ER Osborne D Hotchkiss RS. Frontline Science: Defects in Immune Function in Patients With Sepsis are Associated With PD-1 or PD-L1 Expression and can be Restored by Antibodies Targeting PD-1 or PD-L1. J Leukoc Biol (2016) 100:1239–54. doi: 10.1189/jlb.4HI0616-255R PMC5110001 27671246

[34] Klein SL Flanagan KL. Sex Differences in Immune Responses. Nat Rev Immunol (2016) 16:626–38. doi: 10.1038/nri.2016.90 27546235

[35] Farzadegan H Hoover DR Astemborski J Lyles CM Margolick JB Markham RB. Sex Differences in HIV-1 Viral Load and Progression to AIDS. Lancet (1998) 352:1510–4. doi: 10.1016/S0140-6736(98)02372-1 9820299

[36] Cook IF. Sexual Dimorphism of Humoral Immunity With Human Vaccines. Vaccine (2008) 26:3551–5. doi: 10.1016/j.vaccine.2008.04.054 18524433

[37] Engler RJM Nelson MR Klote MM VanRaden MJ Huang C-Y Cox NJ. Half- vs Full-Dose Trivalent Inactivated Influenza Vaccine (2004-2005): Age, Dose, and Sex Effects on Immune Responses. Arch Internal Med (2008) 168:2405–14. doi: 10.1001/archinternmed.2008.513 19064822

[38] Critchley HOD Maybin JA Armstrong GM Williams ARW. Physiology of the Endometrium and Regulation of Menstruation. Physiol Rev (2020) 100:1149–79. doi: 10.1152/physrev.00031.2019 32031903

[39] Benjelloun F Quillay H Cannou C Marlin R Madec Y Fernandez H. Activation of Toll-Like Receptors Differentially Modulates Inflammation in the Human Reproductive Tract: Preliminary Findings. Front Immunol (2020) 11:1655. doi: 10.3389/fimmu.2020.01655 32849571 PMC7417306

[40] Guterstam YC Strunz B Ivarsson MA Zimmer C Melin A-S Jonasson AF. The Cytokine Profile of Menstrual Blood. Acta Obstetricia Gynecol Scandinavica (2021) 100:339–46. doi: 10.1111/aogs.13990 PMC7891423 32892344

[41] Al-Harthi L Wright DJ Anderson D Cohen M Matityahu D Cohn J. The Impact of the Ovulatory Cycle on Cytokine Production: Evaluation of Systemic, Cervicovaginal, and Salivary Compartments. J Interferon Cytokine Res (2000) 20:719–24. doi: 10.1089/10799900050116426 10954915

[42] Kyongo JK Jespers V Goovaerts O Michiels J Menten J Fichorova RN. Searching for Lower Female Genital Tract Soluble and Cellular Biomarkers: Defining Levels and Predictors in a Cohort of Healthy Caucasian Women. PloS One (2012) 7:e43951. doi: 10.1371/journal.pone.0043951 22952818 PMC3432048

[43] Francis SC Hou Y Baisley K van de Wijgert J Watson-Jones D TT A. Immune Activation in the Female Genital Tract: Expression Profiles of Soluble Proteins in Women at High Risk for HIV Infection. PloS One (2016) 11:e0143109. doi: 10.1371/journal.pone.0143109 26814891 PMC4729472

[44] Lemaitre J Cosma A Desjardins D Lambotte O Le Grand R. Mass Cytometry Reveals the Immaturity of Circulating Neutrophils During SIV Infection. J Innate Immun (2020) 12:170–81. doi: 10.1159/000499841 PMC7103026 31230057

[45] Evrard M Kwok IWH Chong SZ Teng KWW Becht E Chen J. Developmental Analysis of Bone Marrow Neutrophils Reveals Populations Specialized in Expansion, Trafficking, and Effector Functions. Immunity (2018) 48:364–79.e8. doi: 10.1016/j.immuni.2018.02.002 29466759

[46] Symons LK Miller JE Tyryshkin K Monsanto SP Marks RM Lingegowda H. Neutrophil Recruitment and Function in Endometriosis Patients and a Syngeneic Murine Model. FASEB J (2020) 34:1558–75. doi: 10.1096/fj.201902272R 31914688

[47] McLoughlin RM Witowski J Robson RL Wilkinson TS Hurst SM Williams AS. Interplay Between IFN-γ and IL-6 Signaling Governs Neutrophil Trafficking and Apoptosis During Acute Inflammation. J Clin Invest (2003) 112:598–607. doi: 10.1172/JCI200317129 12925700 PMC171385

[48] Balamayooran G Batra S Balamayooran T Cai S Pacher P Jeyaseelan S. Intrapulmonary G-CSF Rescues Neutrophil Recruitment to the Lung and Neutrophil Release to Blood in Gram-Negative Bacterial Infection in MCP-1–/– Mice. J Immunol (2012) 189:5849–59. doi: 10.4049/jimmunol.1200585 PMC3518636 23129755

[49] Laan M Prause O Miyamoto M Sjöstrand M Hytönen AM Kaneko T. A Role of GM-CSF in the Accumulation of Neutrophils in the Airways Caused by IL-17 and TNF-Alpha. Eur Respir J (2003) 21:387–93. doi: 10.1183/09031936.03.00303503 12661990

[50] Nowak J Borkowska B Pawlowski B. Leukocyte Changes Across Menstruation, Ovulation, and Mid-Luteal Phase and Association With Sex Hormone Variation. Am J Hum Biol (2016) 28:721–8. doi: 10.1002/ajhb.22856 27088641

[51] Armstrong GM Maybin JA Murray AA Nicol M Walker C Saunders PTK. Endometrial Apoptosis and Neutrophil Infiltration During Menstruation Exhibits Spatial and Temporal Dynamics That Are Recapitulated in a Mouse Model. Sci Rep (2017) 7:17416. doi: 10.1038/s41598-017-17565-x 29234102 PMC5727295

[52] Kaur H Merchant M Haque MM Mande SS. Crosstalk Between Female Gonadal Hormones and Vaginal Microbiota Across Various Phases of Women’s Gynecological Lifecycle. Front Microbiol (2020) 11:551. doi: 10.3389/fmicb.2020.00551 32296412 PMC7136476

[53] Jarosik GP Land CB Duhon P Chandler R Mercer T. Acquisition of Iron by Gardnerella Vaginalis. Infect Immun (1998) 66:5041–7. doi: 10.1128/IAI.66.10.5041-5047.1998 PMC108627 9746616

[54] Marx PA Spira AI Gettie A Dailey PJ Veazey RS Lackner AA. Progesterone Implants Enhance SIV Vaginal Transmission and Early Virus Load. Nat Med (1996) 2:1084–9. doi: 10.1038/nm1096-1084 8837605

[55] Hickey DK Patel MV Fahey JV Wira CR. Innate and Adaptive Immunity at Mucosal Surfaces of the Female Reproductive Tract: Stratification and Integration of Immune Protection Against the Transmission of Sexually Transmitted Infections. J Reprod Immunol (2011) 88:185–94. doi: 10.1016/j.jri.2011.01.005 PMC3094911 21353708

