# Supplementary figures and images for: Local Innate Markers and Vaginal Microbiota Composition Are Influenced by Hormonal Cycle Phases
Source: Front Immunol. 2022 Mar 25;13:841723. doi: 10.3389/fimmu.2022.841723 (PMC8990777; doi:10.3389/fimmu.2022.841723)

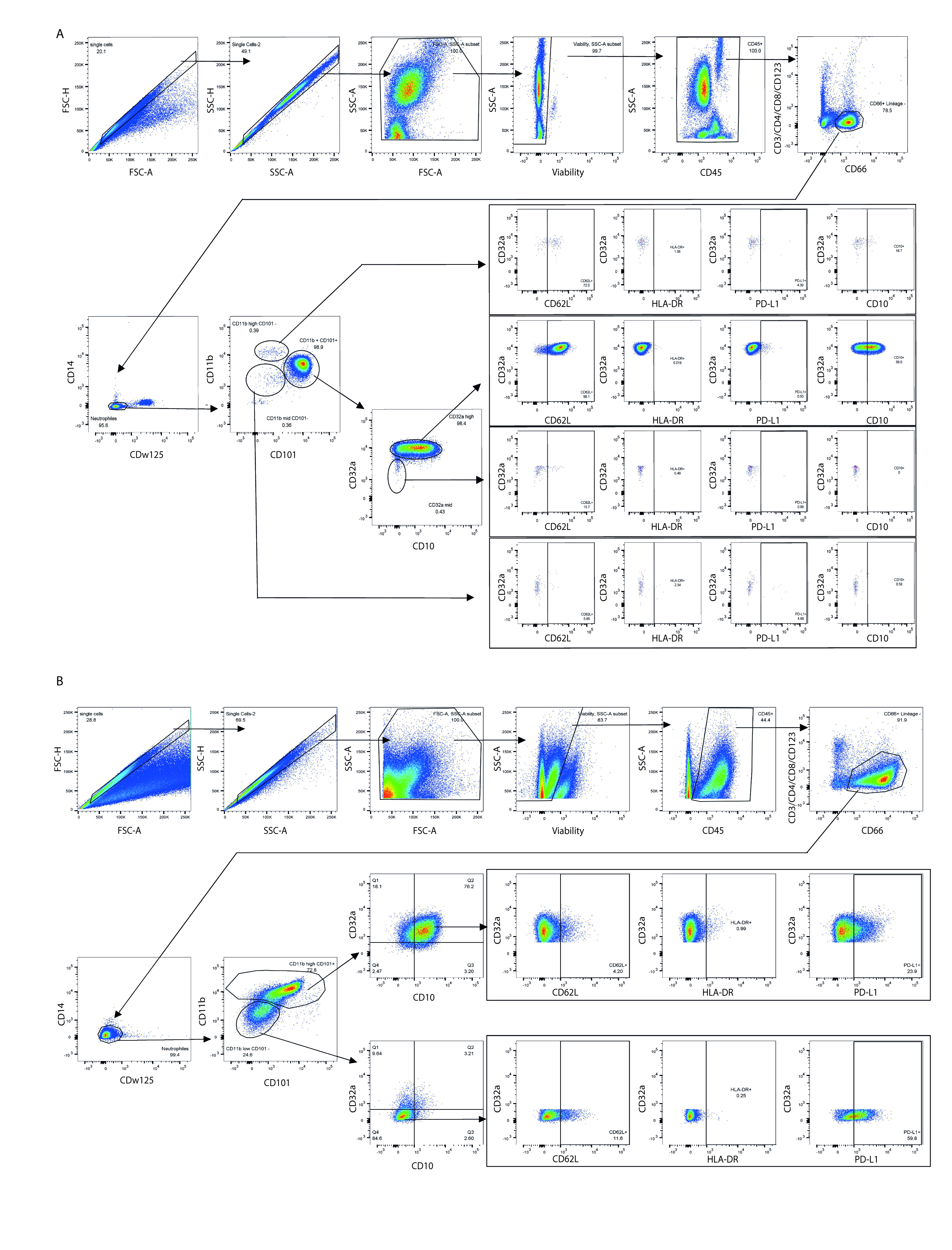

Supplement: Supplementary Figure 1 — FACS gating stategy for neutrophil phenotyping in blood samples (A) and cervicovaginal cytobrushes (B) of one representative animal. [file Image_1.jpeg]

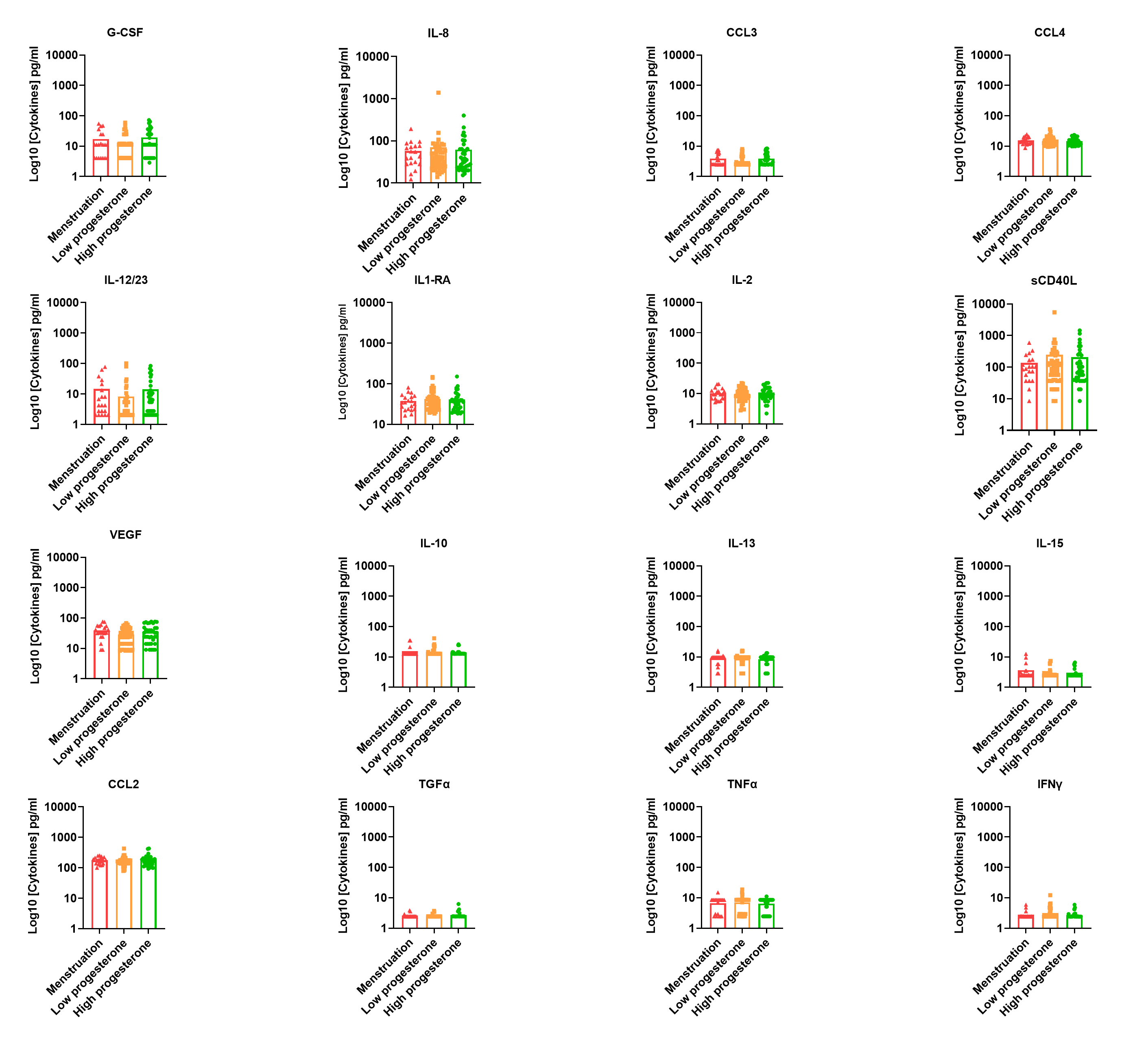

Supplement: Supplementary Figure 2 — Cytokine and chemokine expression in the plasma of female cynomolgus macaques according to the phase of the hormonal cycle (n = 9). Samples were clustered into three groups based on progesterone level or menstruation and each cytokine/chemokine concentration (pg/mL) was plotted. A Kruskal-Wallis test followed by Dunn’s test to adjust the p value was performed. [file Image_2.tif]

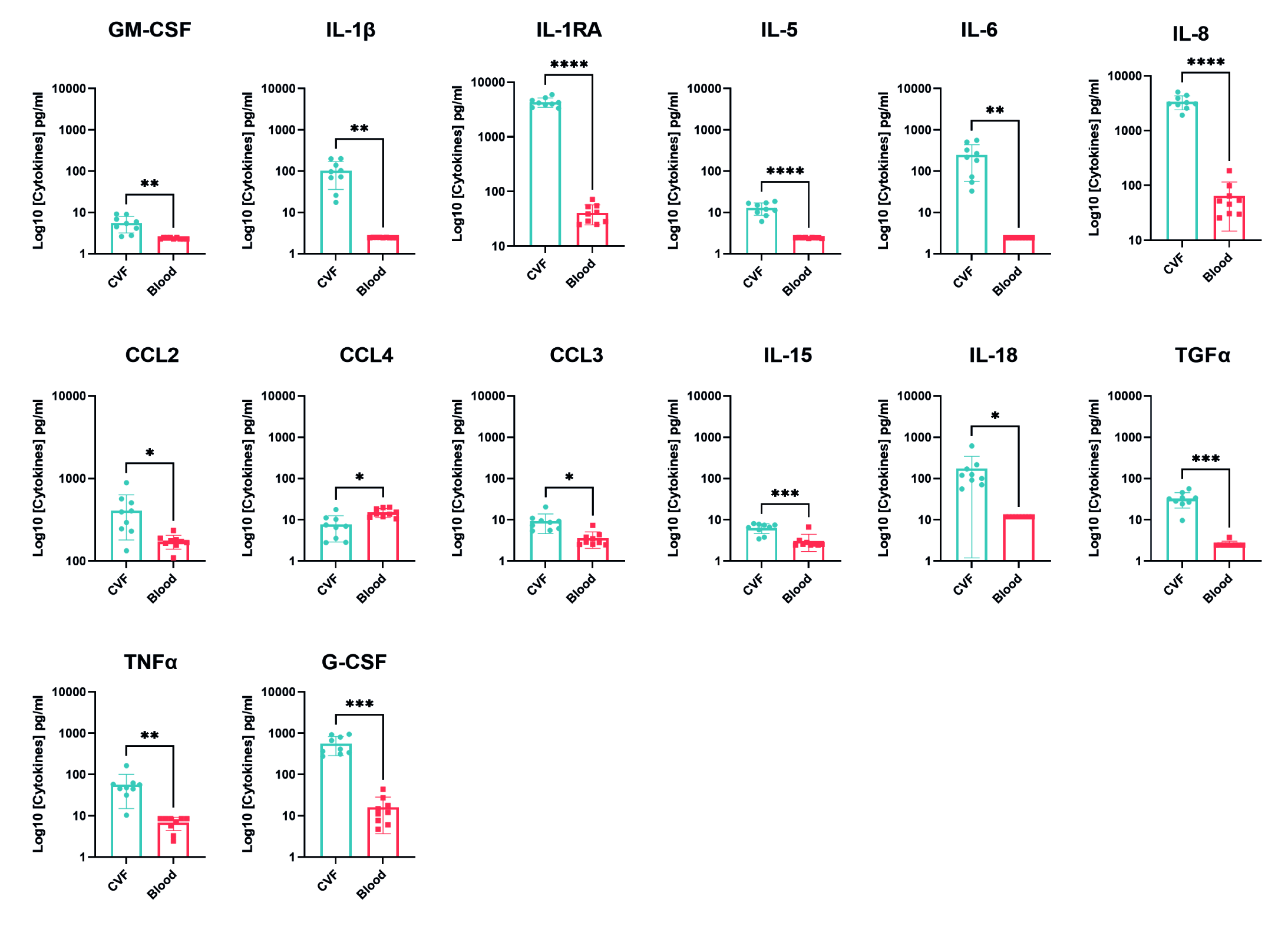

Supplement: Supplementary Figure 3 — Cytokine and chemokine expression in the plasma and cervicovaginal fluids (CVF) of female cynomolgus macaques. The mean concentration of each cytokine/chemokine for each animal in the plasma and CVF were plotted and a paired T-test was performed to compare the two compartments. Asterisks indicate p values considered to be statistically significant (*p ≤ 0.05, **p ≤ 0.01, ***p ≤ 0.001, ****p ≤ 0.0001). [file Image_3.tif]

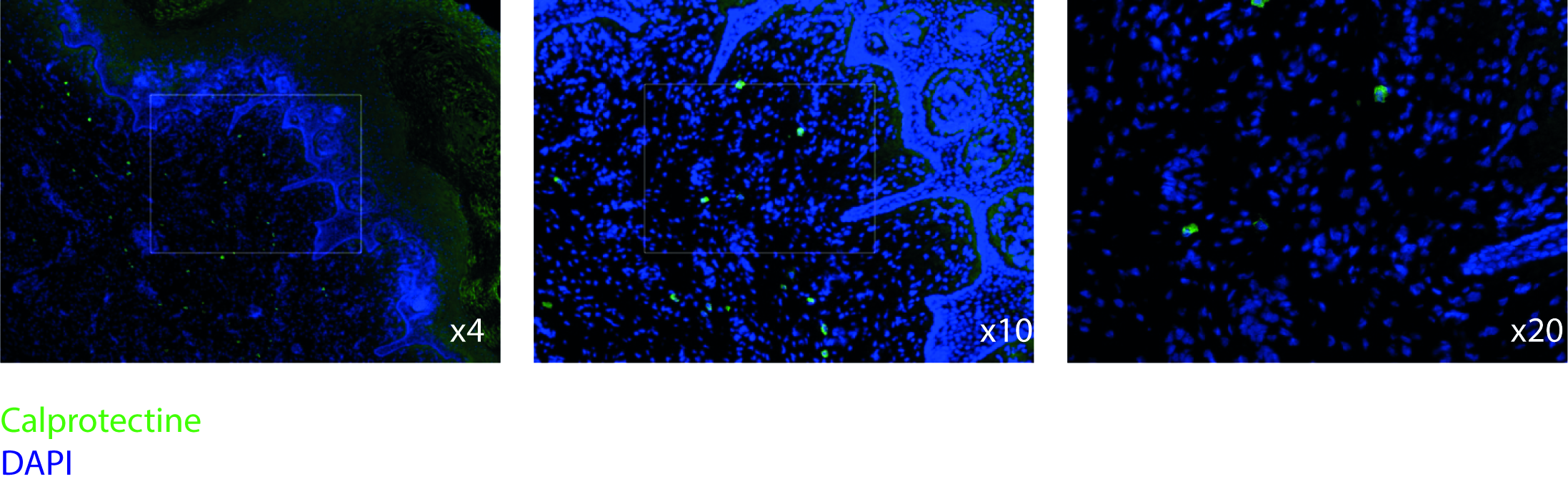

Supplement: Supplementary Figure 4 — Localization of neutrophils in the vaginal mucosa. Immunohistochemistry staining of a slide obtained from a vaginal punch (upper vagina near the cervix) of one female cynomolgus macaque. An anti-calprotectin antibody labelled with FITC (green) was used to stain neutrophils and DAPI to stain the nucleus (blue). [file Image_4.tif]

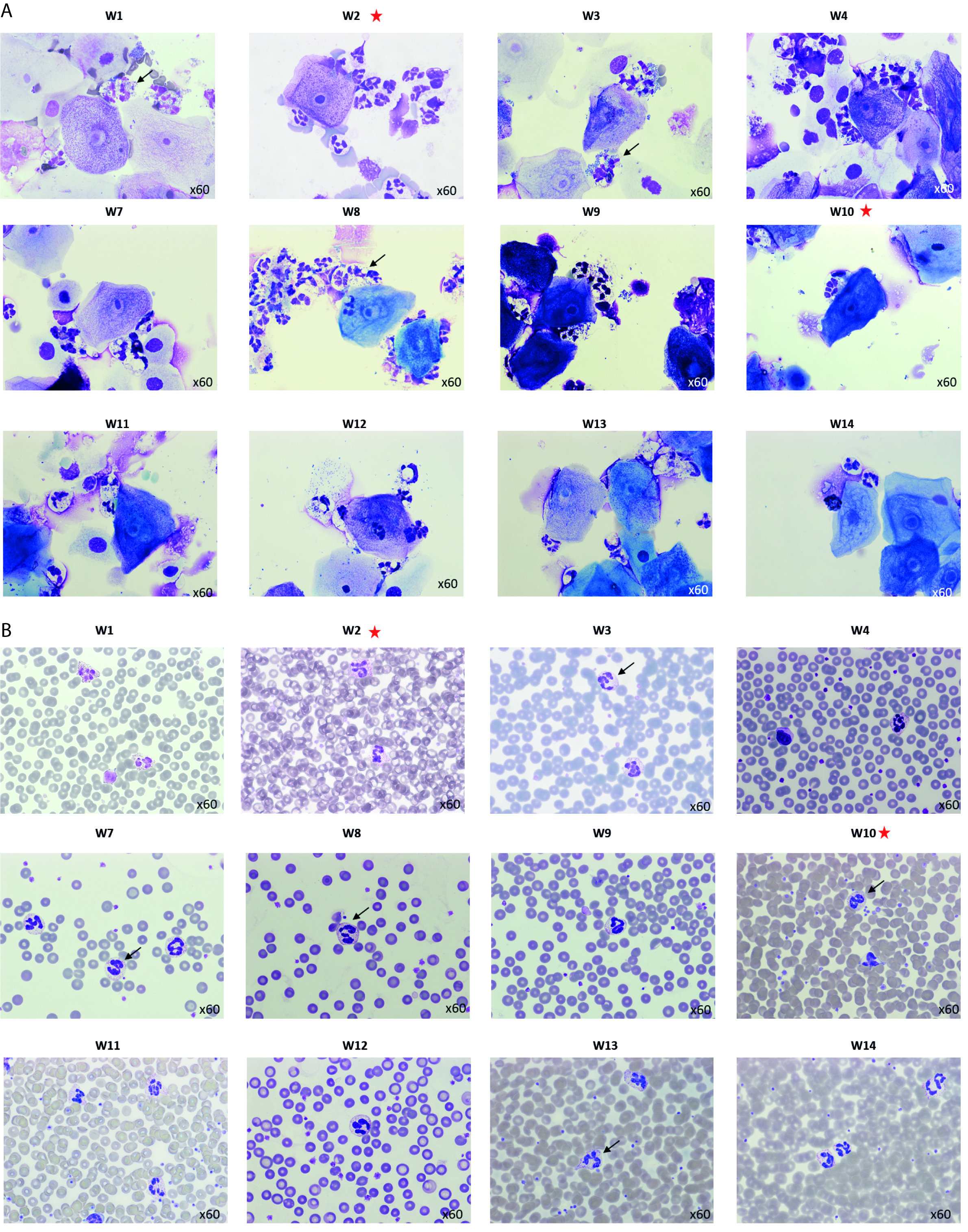

Supplement: Supplementary Figure 5 — Neutrophil morphology in the blood and cervicovaginal cytobrushes. Neutrophil staining by May-Grunwald-Giemsa of (A) cervicovaginal cells obtained from cytobrushes and (B) blood smears of one representative animal (MF7) for all time points. The red star represents menstruation and the black arrow neutrophils. [file Image_5.jpeg]

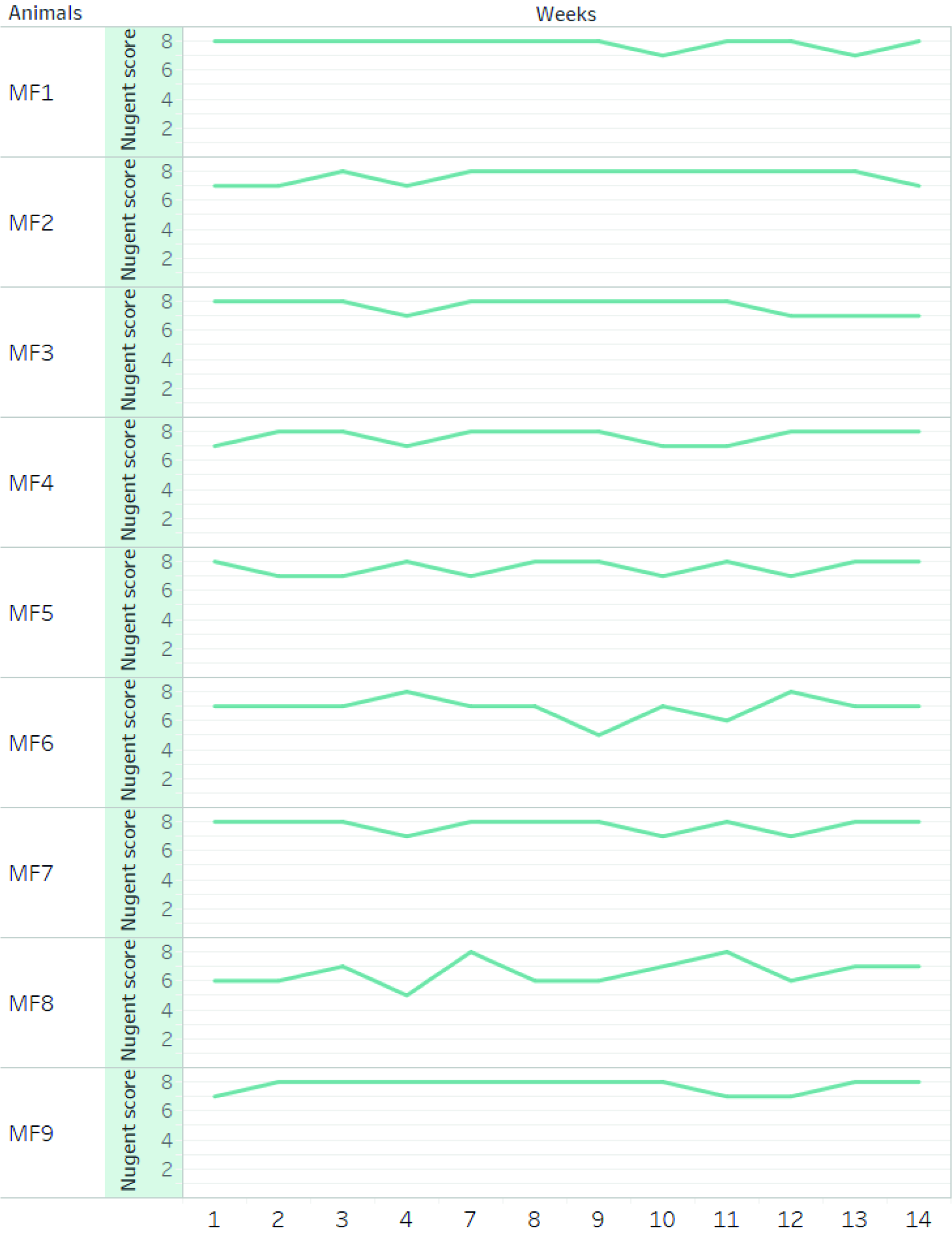

Supplement: Supplementary Figure 6 — Nugent scores for each female during the three months follow-up. [file Image_6.tif]

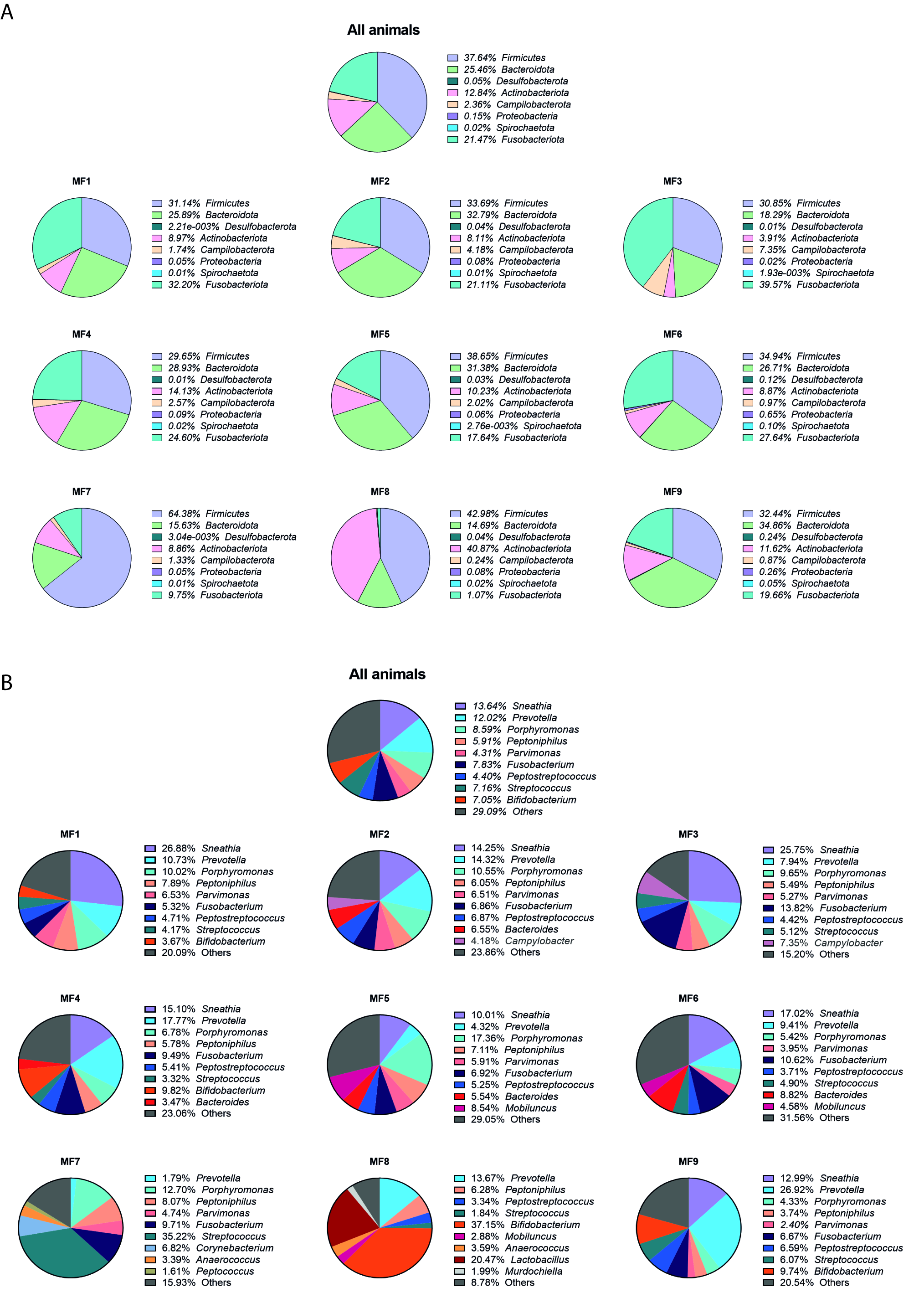

Supplement: Supplementary Figure 7 — Vaginal microbiota composition of the female cynomolgus macaques (n = 9). The percentage of the mean relative abundance of phyla (A) or the top nine most represented genera (B) for all animals (top) or for each female (bottom) are represented in the pie chart. [file Image_7.tif]

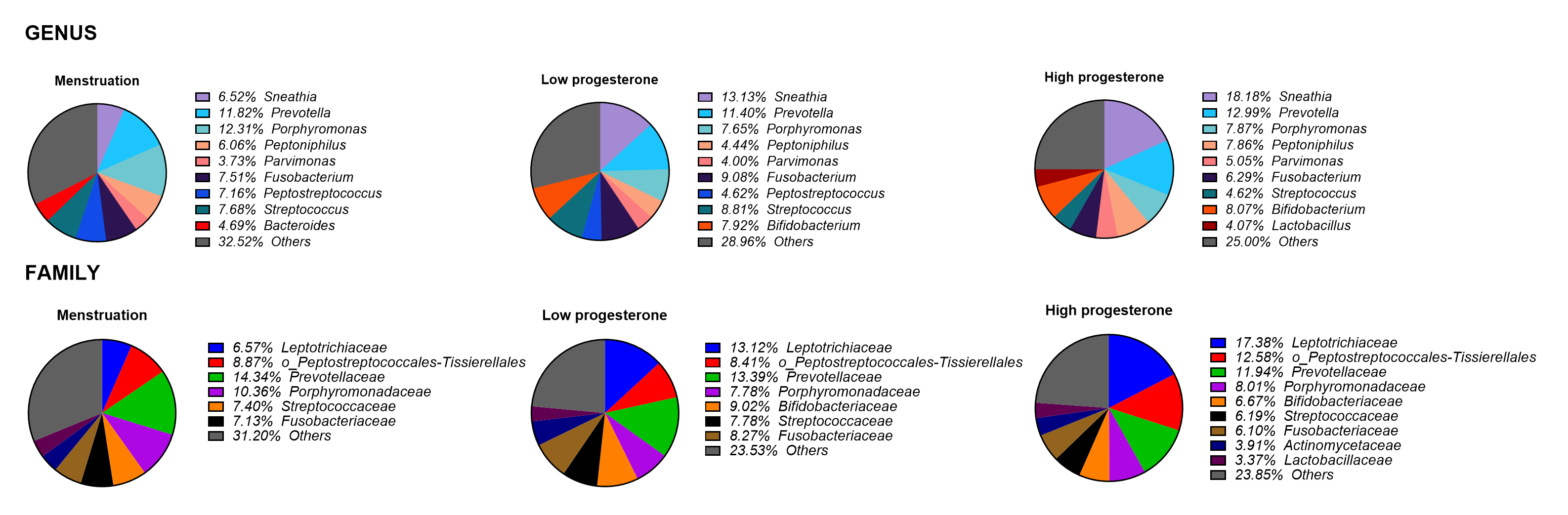

Supplement: Supplementary Figure 8 — Variation of the abundance of bacterial taxa in each phase of the menstrual cycle. The mean relative abundance of the nine most represented genera (top) and families (below) in the high-progesterone, low-progesterone, and menstruation groups is represented in the pie chart for all females. Other genera are shown in grey (other). [file Image_8.tif]

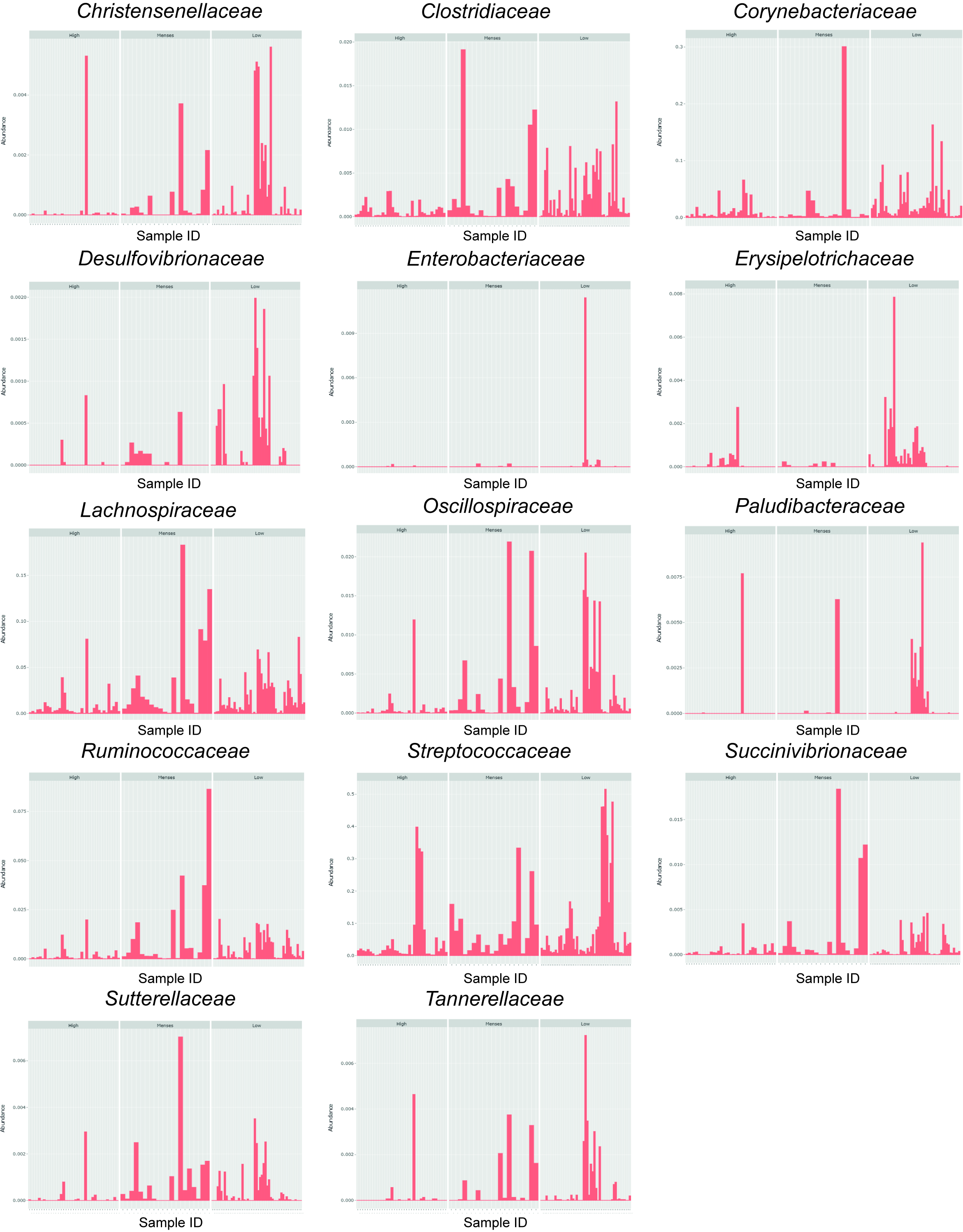

Supplement: Supplementary Figure 9 — Graphical representation of the relative abundance of bacterial taxa according to the hormonal phase at the family level. Only bacterial taxa that were differentially expressed according to the hormonal phase are represented. [file Image_9.jpeg]

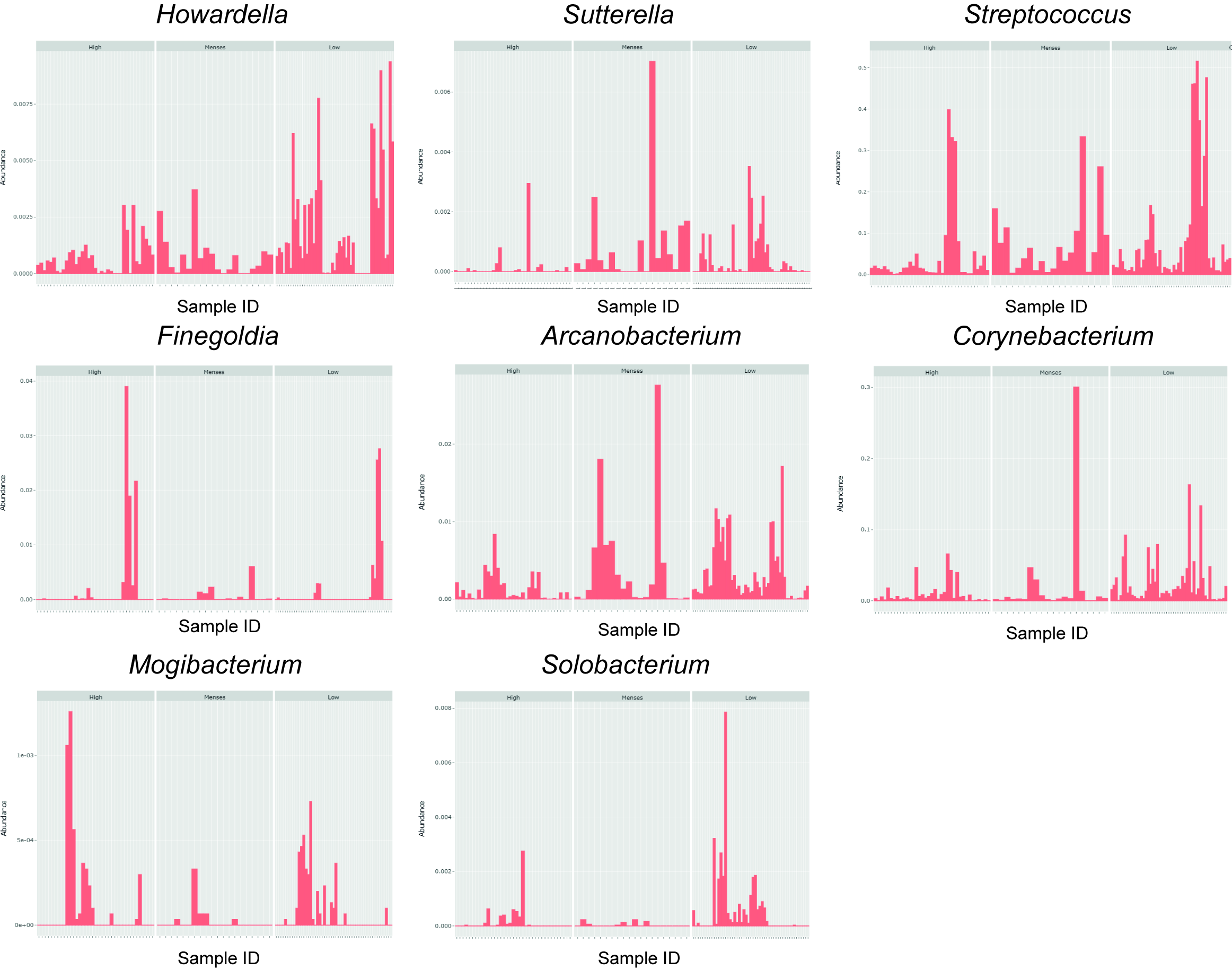

Supplement: Supplementary Figure 10 — Graphical representation of the relative abundance of bacterial taxa according to the hormonal phase at the genus level. Only bacterial taxa that were differentially expressed according to the hormonal phase are represented. [file Image_10.tif]
